# Clinical Evidence of Orally Administered Herbal Preparations for Supportive Cancer Care: A PRISMA Systematic Review of Randomized Clinical Trials (1990–2026)

**DOI:** 10.3390/medsci14060597

**Published:** 2026-09-22

**Authors:** Rebeca Moreno-Ponce, Uriel Díaz-Llerenas, Gustavo A. Hernández-Fuentes, Idalia Garza-Veloz, Dalila G. Virgen-Aguilar, Mario A. Alcalá-Pérez, Emmanuel Vallejo-Tapia, Karla B. Carrazco-Peña, Karmina Sánchez-Meza, Víctor H. Cervantes-Kardasch, Mario Del-Toro-Equihua, Nancy A. Reyes-Méndez, Gael E. Gudiño-Ramírez, Margarita de la Luz Martinez-Fierro, Iván Delgado-Enciso

**Affiliations:** 1 Faculty of Chemical Sciences, University of Colima, Colima 28400, Mexico; rmoreno12@ucol.mx (R.M.-P.);; 2 Molecular Medicine Laboratory, Academic Unit of Human Medicine and Health Sciences, Autonomous University of Zacatecas, Carretera Zacatecas-Guadalajara Km. 6, Ejido la Escondida, Zacatecas 98160, Mexico; 3 Department of Molecular Medicine, Faculty of Medicine, University of Colima, Colima 28040, Mexico; 4 State Cancerology Institute of Colima, Health Services of the Mexican Social Security Institute for Welfare (IMSS-BIENESTAR), Colima 28085, Mexico; 5 Carrera de Medicina Integral y Salud Comunitaria, Sede Armería, Universidad para el Bienestar Benito Juárez García (UBBJ), Degollado 428, Ejido Armería, Colima 28305, Mexico; 6 Health Department, El Colegio de la Frontera Sur, San Cristóbal de las Casas, Chiapas 29290, Mexico; nancy.reyes@posgrado.ecosur.mx; 7 Department of Dietetics and Nutrition, Robert Stempel College of Public Health and Social Work, Florida International University, Miami, FL 33199, USA

**Keywords:** herbal preparations, herbal infusions, supportive cancer care, medicinal plants, complementary oncology, phytotherapy, randomized controlled trials, systematic review

## Abstract

Background/Objectives: Orally administered herbal preparations have gained attention as complementary approaches in supportive cancer care; however, their clinical efficacy and safety remain uncertain. This systematic review critically evaluated evidence from randomized clinical trials investigating these interventions in patients with cancer. Methods: The review followed Preferred Reporting Items for Systematic Reviews and Meta-Analyses (PRISMA) 2020 guidelines and was prospectively registered in PROSPERO. PubMed/MEDLINE, ScienceDirect, Springer Nature Link, CENTRAL, Scopus, LILACS, ClinicalTrials.gov, and WHO ICTRP were searched for English-language studies published from January 1990 through 8 September 2026. Risk of bias was assessed using RoB 2, and outcome-level certainty was evaluated using GRADE. Results: Eight randomized clinical trials met the eligibility criteria and were included in the qualitative synthesis. The studies evaluated Gamiguibi-tang, Rikkunshito, holy basil (*Ocimum tenuiflorum*), *Alcea digitata*–*Malva sylvestris*, *Annona muricata*, *Malva sylvestris*–*Althaea digitata*, Jing Si herbal tea, and Renshen Yangrong Tang across heterogeneous cancer populations. Individual trials reported potentially favorable findings for sleep, fatigue, chemotherapy-induced anorexia, anxiety, salivary function, xerostomia, gastrointestinal symptoms, and quality of life. However, the Jing Si herbal tea trial found no statistically significant between-group differences in fatigue or quality of life, and exploratory ex vivo cytotoxicity findings did not demonstrate clinical antitumor efficacy. Meta-analysis was not feasible because of substantial clinical and methodological heterogeneity. All evaluated outcomes were rated as very-low-certainty evidence, primarily because of risk of bias and serious imprecision. No serious intervention-related adverse events were identified in the published reports, but adverse-event monitoring was inconsistent and no trial was adequately powered to establish safety. Conclusions: The available evidence is insufficient to establish definitive efficacy or safety or to support routine clinical implementation. Adequately powered multicenter trials using botanically authenticated and chemically characterized preparations, clinically meaningful outcomes, interaction assessment, and long-term pharmacovigilance are required.

## 1. Introduction

Cancer is one of the leading causes of morbidity and mortality worldwide, representing a major challenge for health systems because of its increasing incidence and its physical, psychological, and economic impact on patients and families. According to GLOBOCAN 2020 estimates, approximately 19.3 million new cancer cases and nearly 10 million cancer deaths were reported in 185 countries, underscoring the magnitude of cancer as a global public health problem [1]. Epidemiological projections further indicate that the global cancer burden will continue to rise in the coming decades, mainly due to population growth, aging, and exposure to multiple risk factors [2].

Despite advances in surgery, radiotherapy, chemotherapy, targeted therapies, and immunotherapy, oncology treatments continue to be associated with adverse effects that may significantly compromise patients’ quality of life. Common symptoms include fatigue, sleep disturbances, anxiety, depression, anorexia, oral mucositis, neuropathies, and other complications derived from both the disease and its treatment [3]. Consequently, supportive oncology has become an essential component of comprehensive cancer care, focusing on the prevention and management of treatment-related toxicities while preserving patients’ quality of life throughout the course of the disease. In this context, complementary therapeutic strategies have attracted increasing interest as potential adjuncts to conventional cancer treatment, particularly those perceived as safe because of their long-standing traditional use. However, long-standing traditional use should not be interpreted as evidence of clinical efficacy or safety.

Within these complementary strategies, orally administered herbal preparations have attracted interest because of their widespread traditional use and their potential role in the management of cancer- or treatment-related symptoms [4]. These preparations may originate from the leaves, flowers, roots, seeds, fruits, bark, or other parts of medicinal plants and may be produced through infusion, decoction, or aqueous extraction [4]. Unlike conventional tea prepared from *Camellia sinensis*, they encompass diverse single-herb and polyherbal preparations used in traditional health systems, including Traditional Chinese Medicine, Kampo medicine, Ayurvedic medicine, and other ethnomedical practices.

Orally administered herbal preparations may contain polyphenols, flavonoids, saponins, polysaccharides, alkaloids, phenolic acids, and other secondary metabolites [5,6]. Experimental studies have associated these constituents with antioxidant, anti-inflammatory, immunomodulatory, cytoprotective, and other biological activities [7,8]. Such mechanisms may provide biological context for clinical outcomes such as fatigue, anorexia, anxiety, xerostomia, gastrointestinal symptoms, and quality of life. However, these properties are derived predominantly from phytochemical and preclinical research and do not establish that orally administered preparations deliver clinically relevant systemic concentrations or produce meaningful patient benefits. Aqueous extraction, gastrointestinal absorption, metabolism, and bioavailability may differ substantially among compounds and formulations.

Despite their natural origin, orally administered herbal preparations should not be assumed to be inherently safe.

Variability in botanical species, plant parts, phytochemical composition, preparation procedures, and dosage, together with the potential for herb–drug interactions, requires rigorous clinical evaluation before these interventions can be recommended as evidence-based supportive therapies. Chemically characterized and quality-controlled phytotherapeutic products should also be distinguished from poorly standardized or unregulated formulations because their reproducibility, efficacy, and safety profiles may differ substantially.

Published randomized clinical trials have evaluated different medicinal plant species, cancer types, intervention protocols, and clinical outcomes, including quality of life, sleep disturbances, fatigue, anxiety, xerostomia, chemotherapy-induced anorexia, and gastrointestinal symptoms. Moreover, most previous reviews have combined heterogeneous pharmaceutical formulations, such as capsules, extracts, granules, dietary supplements, topical preparations, and injectable products, making it difficult to determine the specific contribution of orally administered preparations originating from infusion, decoction, or aqueous-extraction practices. A formulation-sensitive evaluation of the clinical evidence is therefore necessary to avoid generalizing findings across chemically and pharmacologically heterogeneous products.

In accordance with the PICOC approach [9] (Population, Intervention, Comparison, Outcomes, and Context), the present systematic review aimed to identify, critically appraise, and synthesize the available evidence addressing the following research question: What clinical evidence from randomized clinical trials is available regarding the efficacy and safety of orally administered infusion-, decoction-, and aqueous-extract-derived herbal preparations in patients with cancer? By focusing on randomized clinical evidence, this review provides a formulation-sensitive assessment of the potential supportive benefits, safety reporting, certainty of evidence, and current evidence gaps associated with these preparations.

## 2. Materials and Methods

### 2.1. Study Design

A systematic literature review was conducted in accordance with the Preferred Reporting Items for Systematic Reviews and Meta-Analyses (PRISMA) 2020 guidelines to identify, critically appraise, and qualitatively synthesize the available clinical evidence regarding orally administered infusion-, decoction-, and aqueous-extract-derived herbal preparations in patients with cancer [10]. The research question was formulated using the PICOC framework [9] (Population, Intervention, Comparison, Outcomes, and Context), providing a structured approach for defining the search strategy, eligibility criteria, and evidence synthesis.

Because the objective of this review was not only to identify eligible studies but also to critically evaluate their methodological quality and summarize their clinical findings, a systematic literature search was combined with a narrative synthesis of the available evidence. A quantitative meta-analysis was not considered appropriate because of the marked clinical and methodological variability observed among the included studies with respect to cancer type, herbal preparation, formulation, dosage regimen, treatment duration, and evaluated outcomes.

To ensure methodological transparency and minimize the risk of bias during the review process, the study protocol was prospectively registered in the International Prospective Register of Systematic Reviews (PROSPERO, registration number: CRD420261438444). The database and registry search was expanded in response to peer review, and this amendment to the original search procedures is reported transparently in the revised manuscript.

### 2.2. Search Strategy

A comprehensive literature search was initially performed in PubMed/MEDLINE, Springer Nature Link, and ScienceDirect. In response to the reviewer’s recommendation, the search was expanded to include the Cochrane Central Register of Controlled Trials (CENTRAL), Scopus, ClinicalTrials.gov, the World Health Organization International Clinical Trials Registry Platform (WHO ICTRP), and LILACS as a relevant regional database. Eligible records published or registered between 1 January 1990, and 8 September 2026, were considered. The final updated search was conducted on 8 September 2026. Embase and Web of Science were also considered because of their relevance to biomedical and multidisciplinary evidence retrieval. However, these subscription-based databases could not be searched because access was unavailable through the authors’ institutions. Their absence was therefore retained as a limitation of the review. PROSPERO registration was used to support protocol transparency and was not considered a substitute for a comprehensive literature search. Only full-text reports published in English were eligible because resources for reliable translation and duplicate verification of non-English publications were unavailable.

The search strategy was developed according to the PICOC framework and adapted to the indexing systems and search functionalities available in each bibliographic database and trial registry [9]. In PubMed/MEDLINE, controlled vocabulary terms (Medical Subject Headings, MeSH), including *Neoplasms* and *Phytotherapy*, were combined with free-text terms related to cancer, herbal infusions, medicinal plants, phytotherapy, and clinical trials. Database-specific combinations of controlled vocabulary, free-text terms, Boolean operators, and available filters were used in CENTRAL, Scopus, and LILACS. Simplified free-text strategies were used in ScienceDirect and Springer Nature Link. ClinicalTrials.gov and WHO ICTRP were searched using combinations of terms related to cancer, herbal preparations, phytotherapy, and clinical intervention studies. The complete strategies, dates, and numbers of records retrieved from each source are presented in Table 1 and the Supplementary Materials. Boolean operators (AND and OR) were used to combine search terms appropriately.

The comprehensive literature search conducted across PubMed/MEDLINE, ScienceDirect, Springer Nature Link, CENTRAL, Scopus, LILACS, ClinicalTrials.gov, and WHO ICTRP identified a total of 7475 records before deduplication. The number of records retrieved from each source and the corresponding database-specific search strategies are provided in Table 1 and the Supplementary Materials.

### 2.3. Study Selection Process

All retrieved references were managed using Zotero version 7 reference-management software (Corporation for Digital Scholarship, Vienna, VA, USA). Owing to export limitations in ScienceDirect, records were downloaded in separate chronological intervals (1990–2018, 2019–2024, and 2025–2026). In Springer Nature Link, discrepancies between the number of displayed and exported records were attributed to duplicate versions and related documents generated by the platform.

Subsequently, all references were imported into a Microsoft Excel database containing bibliographic information, including title, authors, year of publication, DOI, database source, and abstract.

After combining the original and expanded search results, 212 duplicate records were removed, including duplicates within the updated searches and records overlapping with the original search. A total of 7263 records remained for title-and-abstract screening.

During title-and-abstract screening, 7226 records were excluded because they were reviews, preclinical or animal studies, evaluated non-cancer populations, investigated ineligible interventions or administration routes, or otherwise failed to meet the predefined eligibility criteria.

A total of 37 reports and trial-registry records were assessed for eligibility by two independent reviewers. Twenty-nine were excluded for the reasons documented in Supplementary Table S2. Eight randomized controlled trials fulfilled the prespecified eligibility criteria and were included in the qualitative synthesis. The Kitchenham-based methodological scores were reported descriptively and were not used as an eligibility threshold [11]. Only full-text reports published in English were eligible.

### 2.4. Eligibility Criteria

No restrictions were imposed regarding participants’ age, sex, cancer type, or clinical stage. Eligible studies were original randomized clinical trials published in English between 1 January 1990, and 8 September 2026, evaluating orally administered plant-based preparations originating from infusion, decoction, or aqueous-extraction practices in patients with a confirmed diagnosis of cancer. Studies were required to report clinical outcomes related to efficacy, safety, or both and to provide sufficient bibliographic and methodological information to permit reliable identification, eligibility assessment, and extraction of clinical outcome data.

Studies were excluded if they evaluated non-cancer populations; non-oral, topical, injectable, or parenteral interventions; preparations that did not meet the prespecified formulation criteria; preclinical, in vitro, or animal experiments; reviews; conference abstracts; books; theses; editorials; study protocols without clinical results; or publications with insufficient information for reliable identification or eligibility assessment.

Study selection was independently performed by two reviewers. Screening was conducted in two sequential stages consisting of title/abstract evaluation followed by full-text assessment. Disagreements were resolved through consensus. Because no quantitative synthesis or meta-analysis was performed, assessment of reporting bias (e.g., funnel plots or small-study effects) was not applicable.

For this review, eligible interventions were operationally defined as orally administered plant-based preparations originating from infusion, decoction, or aqueous-extraction practices. Eligible formulations included freshly prepared infusions or decoctions, aqueous traditional formulations, granules dissolved in water before administration, and aqueous-extract-derived preparations administered orally. Preparations containing isolated compounds, nonaqueous extracts without a documented relationship to an aqueous preparation, topical products, mouthwashes, injectable formulations, and parenteral preparations were excluded. Because formulation can substantially influence phytochemical composition, bioavailability, and safety, differences among the eligible preparations were explicitly considered during evidence synthesis.

### 2.5. Data Extraction

Data extraction was independently performed by two reviewers using a standardized Microsoft Excel data extraction form specifically designed for this review.

Extracted variables included bibliographic information (title, authors, DOI, publication year, journal, and database source), clinical characteristics (cancer type, disease stage, intervention, route of administration, treatment duration, dosage regimen), efficacy outcomes, adverse events, and the principal conclusions reported by each study.

Whenever discrepancies were identified during data extraction, the original publication was re-examined jointly by both reviewers until consensus was achieved, ensuring consistency and accuracy of the extracted information. Effect estimates were extracted and reported as presented in the original trials, including between-group mean differences, changes from baseline, relative effect measures, confidence intervals, and *p*-values, when available. No common summary effect measure was calculated because clinically comparable intervention–outcome groups could not be established.

### 2.6. Descriptive Methodological Assessment and Risk-of-Bias Evaluation

The methodological and reporting characteristics of the 35 published reports with sufficient information for evaluation were descriptively assessed using a structured matrix adapted from Kitchenham and Charters [11] and modified for the objectives of this review. The two trial-registry records without associated publications reporting clinical results were not suitable for this descriptive assessment. The adapted instrument assessed four methodological domains: study design, study conduct, data analysis, and reporting of conclusions. An additional criterion evaluated whether the botanical species was adequately identified and whether the intervention met the prespecified botanical, formulation, and oral-administration criteria.

Each methodological criterion was scored dichotomously as 1 when the criterion was fulfilled and 0 when it was not fulfilled. For descriptive purposes, the criterion addressing botanical identification and conformity with the predefined intervention scope retained a five-point weighting, resulting in a maximum score of 15 points. This weighting was not used as an eligibility requirement or methodological threshold. The Kitchenham-based instrument was applied descriptively to the 35 published reports with sufficient information for evaluation to characterize their methodological and reporting features. It was not used to determine study eligibility, establish a minimum methodological threshold, or assess risk of bias. Inclusion in the qualitative synthesis was based exclusively on fulfillment of the predefined clinical eligibility criteria described in Section 2.4. Quality scores are reported to contextualize the evidence base but were not used as an exclusion threshold. Accordingly, no minimum score constituted a “pass,” and a higher Kitchenham-based score should not be interpreted as indicating a low risk of bias.

Because all studies included in the final qualitative synthesis were randomized clinical trials, their internal validity was additionally assessed using the revised Cochrane Risk of Bias tool for randomized trials (RoB 2). The assessment considered the following five domains: bias arising from the randomization process; bias due to deviations from the intended interventions; bias due to missing outcome data; bias in measurement of the outcome; and bias in selection of the reported result.

Judgments for each RoB 2 domain and the overall risk of bias were classified as low risk of bias, some concerns, or high risk of bias, in accordance with the RoB 2 guidance. The assessment was performed at the level of the principal clinical outcome evaluated in each included trial. Both the descriptive Kitchenham-based assessment and the RoB 2 evaluation were conducted independently by two reviewers. Disagreements were resolved through discussion and consensus; when consensus could not initially be reached, a third reviewer was consulted.

The Kitchenham-based instrument was used exclusively for the descriptive assessment of methodological and reporting characteristics and did not determine eligibility or risk of bias. Eligibility for the qualitative synthesis was determined exclusively according to the prespecified criteria described in Section 2.4. RoB 2 was applied to the principal clinical outcomes of the eight included randomized controlled trials, whereas GRADE was used to assess outcome-level certainty of evidence. Kitchenham-based scores, RoB 2 judgments, and GRADE certainty ratings were evaluated and reported separately and were not combined into a single summary score. The descriptive methodological assessment is presented in Supplementary Table S1, the GRADE Summary of Findings in Supplementary Table S3, and the RoB 2 assessments in Supplementary Table S4 and Supplementary Figure S2.

## 3. Results

### 3.1. Selection of Studies

The comprehensive literature search of PubMed/MEDLINE, ScienceDirect, Springer Nature Link, CENTRAL, Scopus, LILACS, ClinicalTrials.gov, and WHO ICTRP identified 7475 records. After removing 212 duplicate records, 7263 records underwent title-and-abstract screening. Of these, 7226 were excluded because they did not meet the prespecified eligibility criteria.

Thirty-seven reports and trial-registry records were assessed for eligibility. Twenty-nine were excluded for the reasons summarized in Figure 1 and detailed in Supplementary Table S2: ineligible or insufficiently documented formulation (*n* = 8), non-oral or topical administration (*n* = 6), ineligible study design (*n* = 12), and protocol or registry record without eligible published results (*n* = 3). Eight randomized controlled trials fulfilled the prespecified eligibility criteria and were included in the qualitative synthesis.

The 29 reports and trial-registry records excluded from the qualitative synthesis represented a broader body of research on plant-derived interventions in oncology. The principal reasons for exclusion were ineligible or insufficiently documented formulations, non-oral or topical administration, ineligible study designs, and protocols or registry records without eligible published results. Solid oral products were not excluded solely because of their final dosage form; eligibility depended on whether the report established that the intervention originated from an infusion, decoction, or aqueous-extraction process. These records were not considered in the synthesis of efficacy or safety because they did not fulfill the prespecified clinical eligibility criteria. A complete list of the excluded reports and trial-registry records, with one primary reason for exclusion assigned to each record, is provided in Supplementary Table S2.

### 3.2. Botanical and Pharmaceutical Characteristics of Studies Assessed at Full Text

To provide a broader overview of research trends in plant-derived interventions for cancer, an additional descriptive analysis was performed using the 35 published reports with sufficient information for botanical and pharmaceutical characterization. The two trial-registry records without associated publications reporting clinical results were not included in this descriptive analysis. Eight randomized controlled trials fulfilled the prespecified eligibility criteria and were included in the qualitative synthesis, whereas 27 published reports were excluded from the synthesis but retained exclusively for the descriptive characterization of research trends. This analysis should not be interpreted as evidence of clinical efficacy or safety.

Overall, substantial variability was observed in the medicinal plant species, plant organs, and pharmaceutical formulations evaluated across the full-text studies. Mixed plant-part formulations represented 22.9% of the characterized reports, whereas root- and rhizome-based preparations accounted for 14.3%. Leaf- and flower-based interventions each represented 8.6%, while bark-, seed-, and whole-plant preparations were reported less frequently. Approximately one-third of the studies could not be assigned to a single botanical organ because the intervention consisted of complex traditional formulations or insufficient botanical information was reported.

Among the medicinal plants investigated, *Panax ginseng*, *Malva sylvestris*, *Alcea digitata*, and *Camellia sinensis* were among the most frequently reported taxa. *Panax ginseng* was represented in four reports after incorporation of the Renshen Yangrong Tang trial, whereas *Malva sylvestris*, *Alcea digitata*, and *Camellia sinensis* were each represented in three reports. *Panax quinquefolius*, *Viscum album*, and *Zingiber officinale* were reported in two reports each. Numerous additional species were represented only once, highlighting the marked botanical diversity and limited replication of individual interventions.

Considerable variability was also observed in pharmaceutical formulation. Granules or powder-sachet formulations represented the most frequently reported dosage form, followed by herbal teas or infusions, oral extracts, and mouthwash or topical oral preparations. Collectively, these findings demonstrate that the available clinical literature encompasses a broad spectrum of botanical interventions, although relatively few reports evaluated orally administered preparations that fulfilled the prespecified formulation criteria. The principal botanical and pharmaceutical characteristics identified among the 35 published reports with sufficient information for characterization are summarized in Table 2. This descriptive analysis was not intended to evaluate efficacy but rather to characterize research trends among studies that reached the full-text assessment stage.

### 3.3. General Characteristics of the Included Studies

The eight randomized clinical trials included in the qualitative synthesis were published between 2017 and 2025 and evaluated orally administered herbal preparations originating from infusion, decoction, dissolved-granule, or aqueous-extraction practices in patients with different types of cancer. Although all interventions were administered orally, substantial diversity was observed regarding the botanical species evaluated, formulation type, treatment duration, cancer type, and clinical objectives.

The studies included patients with gastrointestinal, breast, lung, colorectal, prostate, head and neck, differentiated thyroid, and gynecologic cancers, as well as cancer survivors with heterogeneous primary diagnoses. The populations encompassed both early-stage and advanced disease and post-treatment survivorship settings. Intervention duration ranged from 4 days to 8 weeks, reflecting differences in therapeutic objectives, including symptom management, supportive care, and treatment-related toxicity reduction.

Overall, the included trials investigated a diverse range of orally administered herbal preparations, including complex traditional polyherbal formulations, water-dissolved extract granules, single- or dual-species infusions, and an aqueous-extract-derived preparation administered in capsules (Table 3).

### 3.4. Types of Orally Administered Herbal Preparations Evaluated

The orally administered herbal preparations included in the selected clinical trials exhibited substantial diversity in botanical composition, preparation method, final dosage form, and therapeutic rationale. The interventions were classified into three broad categories: complex traditional polyherbal formulations, single- or dual-species infusions, and aqueous-extract-derived oral preparations.

Four studies evaluated traditional Asian polyherbal formulations: Gamiguibi-tang (GGBT), Rikkunshito, Jing Si herbal tea (JSHT), and Renshen Yangrong Tang (RSYRT). JSHT is an eight-herb liquid preparation that was evaluated for cancer-related fatigue in patients with gynecologic cancer. However, the trial did not provide trial-specific chromatographic fingerprinting or quantitative marker-compound analysis. RSYRT was evaluated as a 12-herb extract-granule formulation administered orally twice daily for cancer-related fatigue [19]. Trial-specific chromatographic fingerprinting, validated marker-compound quantification, and batch-level chemical characterization were not clearly reported. These formulations contain multiple medicinal plant species traditionally used for constitutional symptoms, appetite, fatigue, and general well-being [12,13,19].

A second group comprised single- or dual-species preparations, including holy basil (*Ocimum tenuiflorum*) tea and formulations based on *Alcea digitata*, *Malva sylvestris*, and *Althaea digitata*, which have traditionally been used because of their anti-inflammatory, demulcent, and mucosal protective properties [14,15,17].

Finally, one study evaluated an oral preparation derived from *Annona muricata* leaves, a medicinal plant that has attracted considerable scientific interest because of its reported phytochemical composition and potential biological activity [16].

Despite the diversity of botanical species and formulations, all interventions were administered orally, in accordance with the predefined eligibility criteria of this systematic review. The observed heterogeneity reflects the current diversity of herbal approaches investigated in supportive oncology and highlights the absence of standardized and chemically comparable herbal preparations across clinical studies.

Gamiguibi-tang and Rikkunshito were classified as complex East Asian polyherbal formulations rather than simple herbal teas. Their inclusion was based on their origin in traditional decoction practices and their administration as aqueous or water-dissolved preparations in the included trials. Their botanical and pharmacological complexity distinguishes them from single-herb infusions; consequently, their findings should not be generalized to conventional herbal teas [12,13]. Jing Si herbal tea was evaluated as an eight-herb liquid preparation. Although its botanical ingredients were described, trial-specific chromatographic fingerprinting and quantitative marker-compound analysis were not reported [18].

### 3.5. Clinical Efficacy and Interpretation of the Included Trials

The eight included trials evaluated heterogeneous herbal preparations as supportive interventions for cancer- or treatment-related symptoms rather than as therapies directly targeting tumor progression. The reported outcomes involved sleep disturbance, fatigue, chemotherapy-induced anorexia, anxiety, salivary function, xerostomia, quality of life, gastrointestinal symptoms, nutritional status, functioning, leukocyte measures, and exploratory ex vivo cytotoxicity [12,13,14,15,16,17,18,19]. However, differences in cancer types, interventions, comparators, outcome measures, and follow-up periods preclude interpretation of these preparations as a homogeneous therapeutic category.

Although statistically significant improvements were reported for selected outcomes, the overall evidence remains limited by small sample sizes, short follow-up periods, methodological limitations, and the absence of clinically interpretable effect estimates in several trials. The principal clinical findings and their interpretation are summarized in Table 4; methodological and reporting characteristics are presented in Table 5; and literature-derived phytochemical constituents and proposed mechanisms are summarized in Table 6. Figure 2 provides an integrated overview of the interventions, proposed mechanisms, and reported outcomes.

To further distinguish statistical significance from potential clinical importance, effect magnitude and interpretability were examined across the included trials. GGBT produced an approximately 5.6-point greater reduction in ISI scores than the wait-list control, whereas the comparatively small improvement in BFI scores raises uncertainty regarding its clinical relevance (Table 4). Rikkunshito attenuated the decline in caloric intake by approximately 7 percentage points, although the clinical importance of this difference remains uncertain. Basil tea was associated with better preservation of salivary-gland function, but the clinical relevance of the anxiety-score changes could not be established because no MCID-based analysis was reported. The *Alcea digitata*–*Malva sylvestris* preparation produced statistically significant but modest differences in xerostomia-related outcomes. In the prostate cancer trial, the approximately 2.3-point reduction in anal-burning VAS scores suggested symptomatic improvement, although RTOG grading did not differ significantly. The *Annona muricata* trial reported exploratory ex vivo cytotoxicity but did not demonstrate clinical antitumor efficacy. Overall, the available findings do not support definitive conclusions or routine clinical implementation. The intervention-by-outcome evidence-gap heatmap further demonstrates that each evaluated relationship was supported by only one RCT, while most outcome domains remain unexamined across interventions (Supplementary Figure S1). The heatmap also integrates the direction of effect, overall RoB 2 judgment, and certainty of evidence, emphasizing the limited and intervention-specific nature of the available findings.

### 3.6. Safety and Adverse Effects

The included trials provided limited short-term tolerability data. Most reported adverse events were mild and self-limiting; however, the small sample sizes, short intervention periods, and inconsistent adverse-event monitoring preclude conclusions regarding either short- or long-term safety.

Mild dyspepsia was reported following treatment with Gamiguibi-tang (GGBT), whereas persistent nausea was observed in some participants receiving holy basil (*Ocimum tenuiflorum*) tea. Participants receiving preparations containing *Alcea digitata* and *Malva sylvestris* reported mild nausea, gastric discomfort, and polyuria, while the *Malva sylvestris*–*Althaea digitata* preparation was associated with mild nausea and abdominal distension. Rikkunshito and the *Annona muricata* leaf preparation were not associated with clinically significant adverse events during the respective intervention periods. The Renshen Yangrong Tang trial described the intervention as tolerable; however, the available safety reporting was insufficient to establish a comparative or long-term safety profile [12,13,14,15,16,17,18,19].

Among the trials reporting adverse events, no severe intervention-related events were identified. However, the limited sample sizes, short intervention periods, and inconsistent adverse-event monitoring preclude definitive conclusions regarding safety. A standardized adverse-event grading system was used in only one of the eight trials [17], laboratory monitoring was only partially reported, and no trial included sufficient post-intervention follow-up to assess delayed or long-term adverse events. None of the eight trials was powered primarily to evaluate safety outcomes. The safety interpretation of orally administered herbal preparations must distinguish poorly characterized or unregulated preparations from standardized, quality-controlled phytomedicines manufactured under GMP conditions, as their quality and risk profiles may differ substantially. In the current debate regarding evidentiary standards for traditional and complementary medicine, Philips et al. argued that clinical integration should require convincing evidence of efficacy and safety, whereas Heinrich emphasized that regulated and quality-assured phytotherapeutic products have an emerging evidence base while acknowledging the need for stronger, higher-quality research [20,21]. The findings of the present review support a cautious, intervention-specific approach. None of the eight trials provided sufficiently complete information on manufacturing standards, batch consistency, regulatory classification, pharmacokinetics, or herb–drug interactions to support generalization of the reported safety findings beyond the specific preparations and conditions evaluated.

### 3.7. Approach to Evidence Synthesis

Individual trials reported potentially favorable findings for sleep quality, cancer-related fatigue, chemotherapy-induced anorexia, anxiety, salivary function, xerostomia, gastrointestinal symptoms, and quality of life. The Renshen Yangrong Tang trial reported a greater short-term reduction in cancer-related fatigue than the low-dose huangqi active-control group. However, the Jing Si herbal tea trial found no statistically significant between-group differences in fatigue or quality of life, and the exploratory ex vivo cytotoxicity outcome reported for *Annona muricata* did not demonstrate clinical antitumor efficacy.

Despite these potentially favorable findings, considerable diversity was observed regarding the botanical species investigated, cancer populations, intervention formulations, comparators, treatment durations, clinical endpoints, and outcome-assessment methods. Consequently, clinically meaningful direct comparisons among trials were not possible, and the evidence was considered unsuitable for quantitative synthesis or meta-analysis.

An outcome-level certainty assessment was conducted using the GRADE approach for the principal outcomes reported across the eight included trials. All evaluated outcomes were rated as very-low-certainty evidence, primarily because each intervention–outcome relationship was supported by a single, generally small trial; most trials had some concerns or high risk of bias according to RoB 2; confidence intervals were frequently unreported; and some outcomes were exploratory, surrogate, or of uncertain clinical relevance. The complete GRADE assessment is presented in Supplementary Table S3.

### 3.8. Methodological Quality Assessment and Risk-of-Bias Evaluation

The principal clinical outcomes of all eight randomized controlled trials included in the qualitative synthesis were assessed using RoB 2. The Kitchenham-based scores were reported separately for descriptive purposes and were not used as an eligibility threshold or as a substitute for RoB 2. Overall, seven trials were judged as having some concerns and one as having a high risk of bias. The principal concerns involved incomplete reporting of random-sequence generation or allocation concealment, deviations from intended interventions, missing outcome data, measurement of subjective outcomes, and insufficient documentation of prespecified analyses.

Reporting of the randomization process varied across trials, and random-sequence generation or allocation-concealment procedures were insufficiently described in several reports. Additional concerns arose from open-label designs, the absence of an indistinguishable placebo, the use of active herbal comparators, and the measurement of subjective outcomes such as anxiety, fatigue, xerostomia, and quality of life. These design characteristics may have influenced participant behavior, adherence, outcome reporting, or measurement.

Missing outcome data raised some concerns in trials that used per-protocol analyses, reported withdrawals, did not analyze all enrolled or randomized participants, or evaluated selected outcomes in participant subsets. Definite evidence of selective reporting was not identified; however, the absence of accessible protocols or prespecified statistical analysis plans for several trials limited definitive assessment of this domain. All eight randomized controlled trials fulfilled the prespecified clinical eligibility criteria, but eligibility should not be interpreted as indicating a low risk of bias. The domain-level RoB 2 judgments are presented in Supplementary Table S4 and Supplementary Figure S2.

## 4. Discussion

This systematic review included eight small and clinically heterogeneous randomized trials evaluating orally administered herbal preparations as supportive interventions in patients with cancer. Individual trials reported potentially favorable findings for sleep, fatigue, chemotherapy-induced anorexia, anxiety, salivary function, xerostomia, gastrointestinal symptoms, and quality of life. However, most intervention–outcome relationships were represented by only one trial, and the recent JSHT trial found no statistically significant between-group improvement in fatigue or quality of life. Therefore, the available evidence should be interpreted as preliminary, intervention-specific, and insufficient to establish definitive clinical efficacy.

The observed clinical findings are biologically plausible and are consistent with previous experimental evidence describing the pharmacological properties of medicinal plants. Talib [22] reported that numerous medicinal plants contain bioactive compounds with antioxidant, anti-inflammatory, and immunomodulatory properties that may contribute to supportive oncology care. Likewise, Tsao and Shen [23,24] highlighted that phenolic compounds, flavonoids, and other secondary metabolites may reduce oxidative stress and modulate inflammatory pathways. Although these mechanisms may partially explain the clinical findings observed in the included studies, the present review was designed to evaluate clinical outcomes rather than mechanistic pathways. Consequently, mechanistic interpretations should be regarded as hypothesis-generating rather than definitive.

Potential matrix effects, polypharmacological actions, and synergistic or antagonistic interactions among phytochemicals may influence the biological activity of complex herbal preparations. However, none of the included RCTs directly evaluated these phenomena. Therefore, they should be considered literature-derived hypotheses rather than demonstrated mechanisms underlying the observed clinical outcomes [22,23,24,25,26].

Importantly, these proposed mechanisms are based primarily on preclinical and literature-derived evidence and were not directly demonstrated by the eight RCTs included in this review. Another important observation emerging from the available evidence is that the reported benefits were primarily related to symptom management and treatment-associated complications rather than direct modification of tumor progression. Gamiguibi-tang (GGBT) was associated with improvements in sleep quality and fatigue, whereas Rikkunshito was associated with a smaller reduction in caloric intake during chemotherapy [12,13].

Holy basil (*Ocimum tenuiflorum*) tea was associated with decreased anxiety and preservation of salivary function [14], while preparations containing *Alcea digitata*, *Malva sylvestris*, and *Althaea digitata* were associated with potentially favorable findings for xerostomia, quality of life, and selected gastrointestinal symptoms [15,17]. In addition, *Annona muricata* leaf preparations were associated with increased markers of cytotoxic activity against colorectal cancer cells while maintaining nutritional status [16]. Nevertheless, this latter finding was derived from a single exploratory clinical trial incorporating ex vivo laboratory analyses and therefore cannot be interpreted as evidence of direct clinical antitumor efficacy or as confirmation of an in vivo mechanism in patients. Li et al. [18] evaluated JSHT for cancer-related fatigue in patients with gynecologic cancer. Although within-group improvements in fatigue measures were reported, no statistically significant between-group differences were observed in fatigue or quality of life. Xu et al. [19] evaluated Renshen Yangrong Tang (RSYRT) in cancer survivors with clinically relevant fatigue and reported a greater short-term reduction in fatigue than with the low-dose huangqi active control. However, the relatively small sample, active herbal comparator, incomplete reporting of allocation concealment and blinding, and absence of a reported 95% confidence interval limit certainty in this finding. These within-group findings do not establish a specific treatment effect and may reflect temporal changes, placebo responses, regression to the mean, or other nonspecific effects. The small sample and short follow-up further limit inference. Overall, some individual trials reported preliminary favorable findings for specific supportive-care outcomes; however, these results were intervention-specific, supported by very-low-certainty evidence, and should not be interpreted as demonstrating a broader role for orally administered herbal preparations in supportive cancer care.

Previous systematic reviews evaluating herbal medicine in oncology have generally concluded that botanical interventions may improve selected cancer-related symptoms. However, these reviews combined multiple pharmaceutical formulations, including capsules, extracts, granules, powders, dietary supplements, topical preparations, and injectable products, precluding formulation-specific conclusions. By applying a formulation-sensitive definition to orally administered infusion-, decoction-, and aqueous-extract-derived herbal preparations, the present review provides a more focused evaluation of this traditional route of administration, thereby reducing formulation-related heterogeneity and facilitating a more clinically relevant interpretation of the available evidence.

This formulation-specific approach is particularly relevant because differences in extraction method, pharmaceutical formulation, phytochemical composition, and bioavailability prevent findings obtained with one herbal preparation from being directly extrapolated to preparations produced through different manufacturing or extraction processes. Despite this focused approach, an important discrepancy remains between clinical practice and the available evidence. Orally administered herbal preparations represent one of the most traditional and widely used forms of medicinal plant administration worldwide, and several observational studies have shown that many patients with cancer consume herbal preparations alongside conventional oncological treatments [27,28]. However, only eight randomized controlled trials fulfilled the prespecified clinical eligibility criteria established for the present review. This marked discrepancy highlights the urgent need for high-quality clinical research capable of generating robust evidence regarding efficacy, safety, and appropriate clinical integration into supportive oncology. To address these limitations systematically, a translational research roadmap is proposed in Supplementary Figure S3. The framework outlines a sequential progression from botanical authentication and chemical standardization through pharmacokinetic and bioavailability assessment, herb–drug interaction and safety testing, adequately powered multicenter RCTs, and long-term pharmacovigilance.

Regarding safety, most included studies reported only mild and self-limiting adverse events, including dyspepsia, nausea, gastric discomfort, polyuria, and abdominal distension, whereas several studies reported no clinically significant intervention-related adverse events. The RSYRT trial also described short-term tolerability, but its safety reporting was insufficient to establish a comparative or long-term safety profile [19]. Nevertheless, the current evidence remains insufficient to establish long-term safety because most trials included relatively small sample sizes, short intervention periods, and inconsistent adverse-event monitoring. Furthermore, patients with cancer frequently receive multimodal therapies, increasing the potential for herb–drug interactions that remain insufficiently investigated [29,30]. Consequently, the available safety data should be interpreted cautiously until confirmed by larger studies with standardized pharmacovigilance procedures.

A fundamental pharmacological question that remains unaddressed by the available clinical evidence is whether orally administered herbal preparations deliver systemically relevant concentrations. Aqueous extraction efficiency is highly compound-dependent, and many bioactive phytochemicals, including poorly water-soluble polyphenols, terpenoids, and alkaloids, may be present in infusions at concentrations insufficient to produce meaningful systemic exposure after oral ingestion, absorption, and first-pass hepatic metabolism. Furthermore, none of the included RCTs incorporated pharmacokinetic measurements or established clinically relevant systemic exposure to specific constituents or their metabolites. Until pharmacokinetic data from adequately designed clinical studies are available, mechanistic interpretations linking specific phytochemical constituents to the observed clinical effects must be considered speculative.

Despite these potentially favorable findings, several methodological limitations reduce the certainty of the available evidence. Only eight randomized controlled trials fulfilled the prespecified clinical eligibility criteria, highlighting the limited availability of rigorous evidence on orally administered herbal preparations in oncology. Eligibility did not imply low risk of bias. The RoB 2 assessment identified concerns related primarily to deviations from intended interventions, incomplete reporting of allocation concealment, missing outcome data, and measurement of subjective outcomes. These methodological limitations reduce confidence in the reported effect estimates and reinforce the need for cautious interpretation of the findings.

Substantial clinical variability was another important limitation. The included studies evaluated different cancer types, disease stages, concomitant oncological treatments, medicinal plant species, preparation methods, administered doses, intervention durations, and outcome measures [10,31]. Consequently, direct comparison among studies was limited and quantitative synthesis was not appropriate [31,32]. Rather than supporting generalized conclusions regarding orally administered herbal preparations as a homogeneous therapeutic category, the available evidence should be interpreted as intervention-specific, with each herbal preparation and formulation requiring independent clinical evaluation before broader conclusions can be established [25,26,33].

Several limitations of the present review warrant consideration. The updated search included PubMed/MEDLINE, ScienceDirect, Springer Nature Link, CENTRAL, Scopus, LILACS, ClinicalTrials.gov, and WHO ICTRP. Nevertheless, Embase and Web of Science could not be searched because institutional access was unavailable. The absence of these subscription-based databases may have resulted in eligible studies being missed and therefore remains a limitation of search completeness. The restriction to English-language publications may also have introduced language bias, particularly because relevant studies of Asian traditional formulations may have been published in other languages. Therefore, some eligible evidence may have been missed, although the direction and magnitude of this effect cannot be determined. Quantitative meta-analysis was not undertaken because clinically meaningful intervention–outcome groups could not be established. After reassessment, quantitative pooling remained inappropriate because each intervention–outcome relationship was generally represented by only one small trial and substantial heterogeneity persisted across herbal preparations, cancer populations, comparators, intervention durations, and outcome measures. The outcome-level GRADE assessment rated the available evidence as very low certainty, primarily because of risk of bias, imprecision, and the availability of only one small trial for each intervention–outcome relationship. Consequently, the findings were synthesized qualitatively and should be interpreted cautiously. The heterogeneity of included interventions, from single-herb infusions to complex Kampo formulations, limited direct comparisons; this scarcity partly reflects the current research trend toward isolating individual bioactive compounds rather than evaluating whole herbal preparations as traditionally consumed. A critical reproducibility limitation was the generally incomplete phytochemical and manufacturing characterization across the eight trials. Plant-to-water ratios, preparation temperatures and times, chromatographic fingerprints, validated marker compounds, and batch-level consistency were frequently not reported. Although one preparation was standardized to a specified acetogenin content and some trials used commercial formulations, these descriptions were not equivalent to comprehensive trial-specific chemical characterization. Additional constraints include international regulatory heterogeneity and small sample sizes with inconsistent adverse-event monitoring, which preclude definitive conclusions regarding long-term safety and herb–drug interactions. These factors should be considered when interpreting the strength and generalizability of the available evidence.

Although the eligibility assessment identified a broader body of clinical research on medicinal plants in oncology, most excluded reports evaluated non-oral or topical interventions, nonaqueous preparations, non-randomized designs, non-oncological populations, or formulations that could not be established as originating from an infusion, decoction, or aqueous-extraction process. These reports did not fulfill the prespecified clinical eligibility criteria and were therefore not included in the efficacy or safety synthesis. An important methodological issue identified across the included studies was the limited phytochemical characterization of the investigated herbal preparations. Although orally administered herbal preparations remain one of the most traditional and widely used forms of medicinal plant administration, substantial variability exists in plant species, plant organs, cultivation conditions, harvesting season, processing, storage, and infusion preparation [34,35]. These factors may considerably influence phytochemical composition and biological activity, thereby limiting reproducibility and direct comparison among clinical studies. Future randomized clinical trials should therefore prioritize standardized orally administered herbal preparations supported by validated chemical marker compounds and chromatographic fingerprinting to improve reproducibility and facilitate comparison across studies [36,37].

The regulatory framework governing orally administered herbal preparations represents another important challenge for their incorporation into evidence-based supportive oncology. Considerable differences exist among countries regarding the classification, quality-control requirements, manufacturing standards, and clinical evidence necessary for orally administered herbal preparations [38,39]. Because many orally administered herbal preparations are prepared outside standardized manufacturing conditions, variability in composition and quality may compromise both reproducibility and patient safety. Accordingly, chemically characterized phytotherapeutic products manufactured under regulated conditions should not be considered evidentially equivalent to poorly standardized or informally prepared formulations, as they may differ substantially in composition, reproducibility, bioavailability, interaction potential, and safety profile [20,21]. Future investigations should employ orally administered herbal preparations manufactured under Good Manufacturing Practice (GMP) conditions [40,41], supported by standardized phytochemical characterization [42,43] and appropriate pharmacovigilance systems to strengthen the quality [44] and reliability of the available evidence [27,28].

A major strength of the present review is the application of highly specific eligibility criteria that restricted inclusion to randomized clinical trials evaluating orally administered herbal preparations in patients with cancer. Unlike previous reviews that combined heterogeneous botanical formulations, this approach reduced some formulation-related heterogeneity and enabled a more formulation-sensitive evaluation. Furthermore, the expanded database and trial-registry search, transparent study-selection process, separate application of RoB 2 and GRADE, and PRISMA-guided reporting strengthen the methodological transparency of the present review.

Overall, the discrepancy between the widespread use of herbal preparations and the limited number of eligible randomized trials underscores the need for adequately powered, multicenter studies employing standardized and chemically characterized interventions, harmonized outcome measures, systematic monitoring of adverse events and herb–drug interactions, and longer follow-up periods. Such studies will be essential to establish the efficacy, long-term safety, and appropriate integration of orally administered herbal preparations into evidence-based supportive oncology [45,46].

## 5. Conclusions

This systematic review identified preliminary favorable findings for specific supportive-care outcomes among eight infusion-, decoction-, or aqueous-extract-derived herbal interventions evaluated in patients with cancer. However, the results varied across interventions and outcomes, and each intervention–outcome relationship was supported by a single small trial with very-low-certainty evidence. Therefore, the current evidence is insufficient to establish definitive efficacy or safety, and the findings cannot be generalized to orally administered herbal preparations as a class. Future research should evaluate each preparation and formulation independently through adequately powered, methodologically rigorous multicenter randomized trials incorporating botanical authentication, chemical standardization, clinically meaningful outcomes, pharmacokinetic and herb–drug interaction assessments, and systematic long-term safety monitoring.

## Figures and Tables

**Figure 1:**
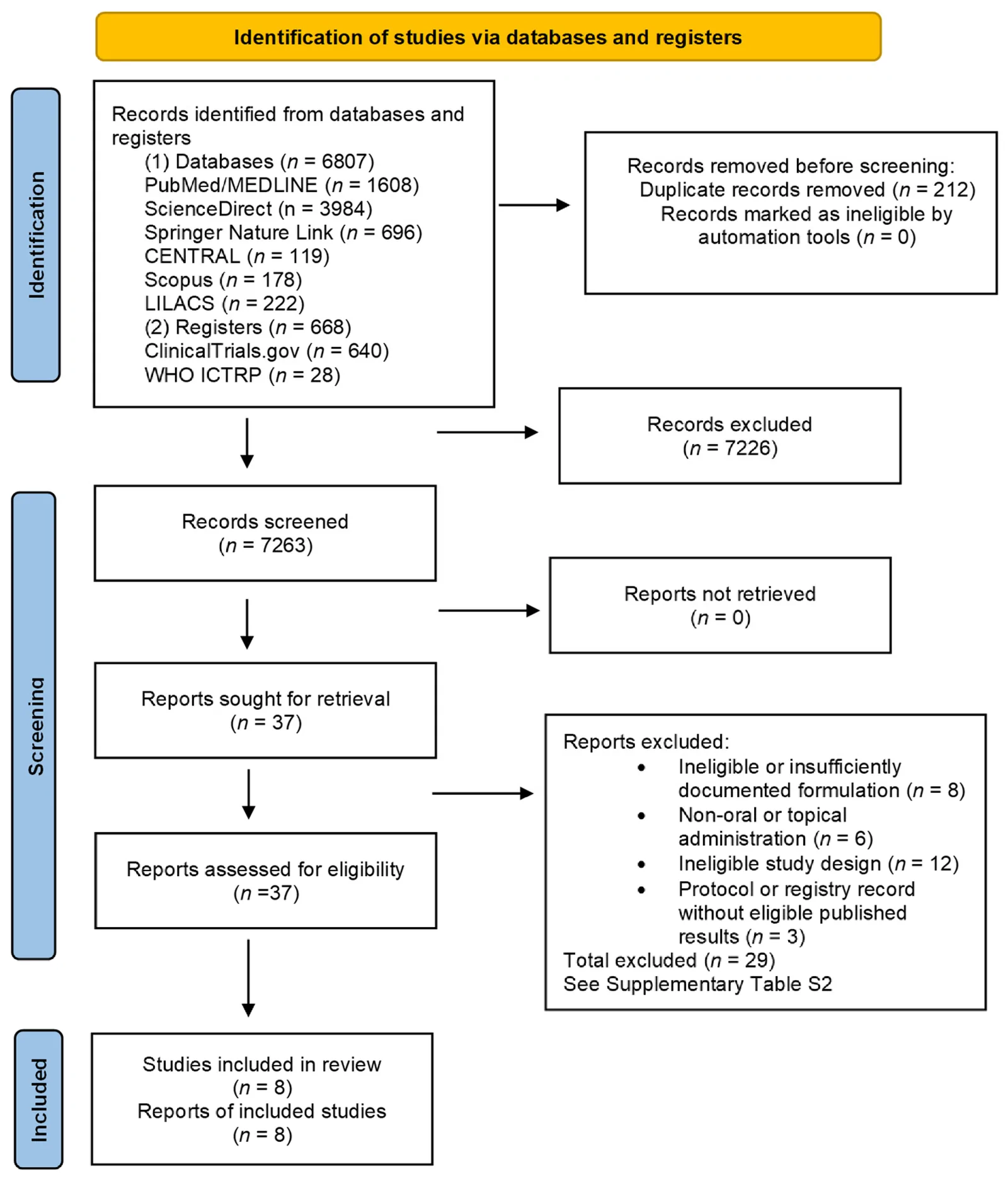
PRISMA 2020 flow diagram of the study-selection process [10]. The comprehensive search of PubMed/MEDLINE, ScienceDirect, Springer Nature Link, CENTRAL, Scopus, LILACS, ClinicalTrials.gov, and WHO ICTRP identified 7475 records. After removing 212 duplicate records, 7263 records underwent title-and-abstract screening, of which 7226 were excluded. Thirty-seven reports and trial-registry records were assessed for eligibility, and 29 were excluded for the reasons summarized in the diagram and detailed in Supplementary Table S2. Eight randomized controlled trials fulfilled the eligibility criteria and were included in the qualitative synthesis. The searches were updated through 8 September 2026. Complete database- and registry-specific search strategies are provided in the Supplementary Materials.

**Figure 2:**
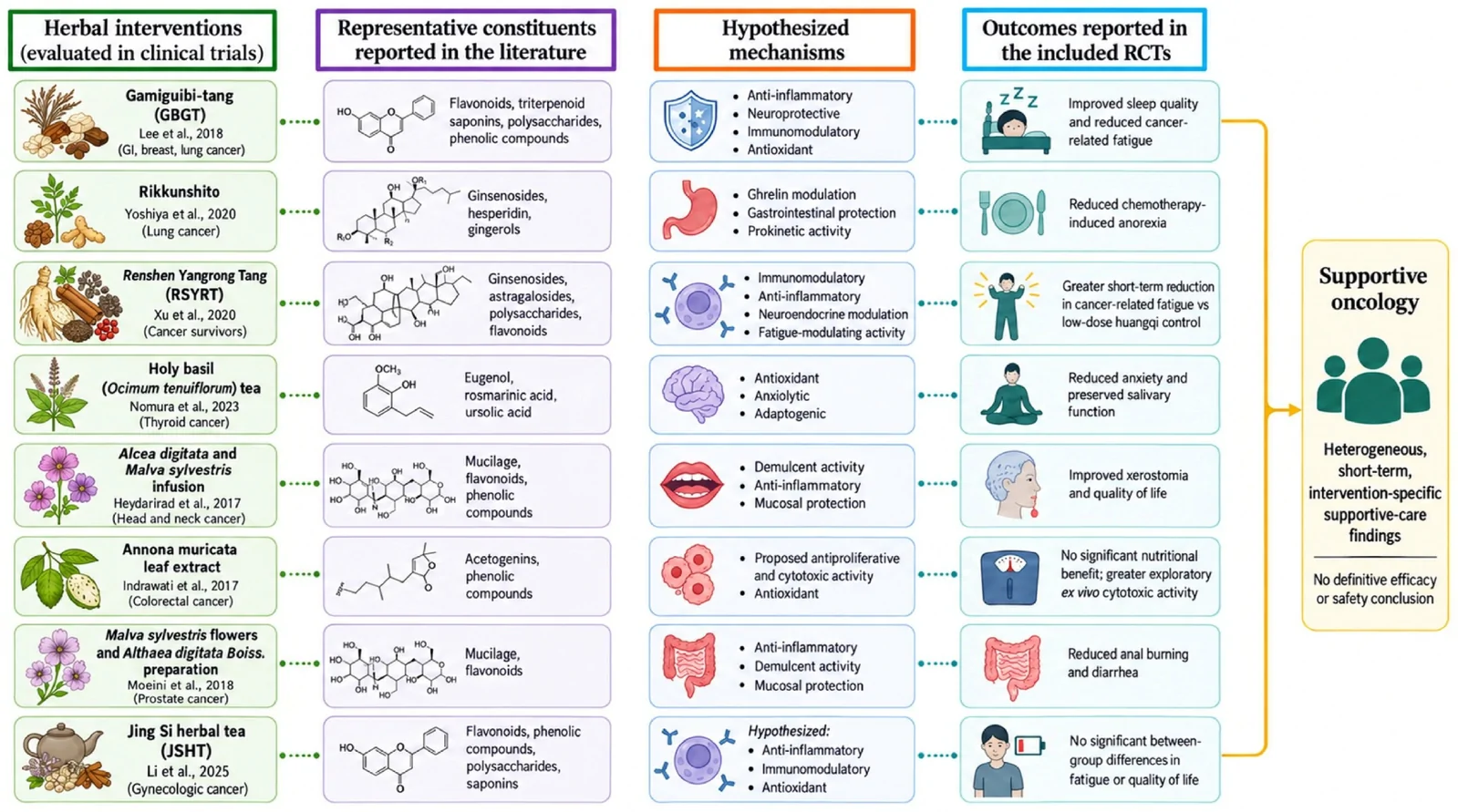
Integrated overview of the orally administered herbal interventions evaluated in the eight included randomized clinical trials, representative phytochemical constituents reported in the literature, hypothesized pharmacological mechanisms, and reported clinical outcomes. The representative constituents and mechanisms were derived from the broader phytochemical, pharmacological, and preclinical literature and were not directly identified, quantified, or mechanistically evaluated in the included RCTs. Accordingly, the connections shown are hypothetical and should not be interpreted as established causal explanations for the reported clinical outcomes. For the representative flavonoid structures, R^1^ may represent OH or H, whereas R^2^ may represent glucose or galactose, illustrating common naturally occurring glycosylated derivatives. For *Annona muricata*, no significant between-group nutritional benefit was demonstrated, while cytotoxic activity was assessed only through an exploratory ex vivo experiment and does not constitute evidence of clinical antitumor efficacy. Xu et al. (2020) [19] reported greater short-term improvement in cancer-related fatigue with Renshen Yangrong Tang than with a low-dose huangqi active control; however, the representative constituents and hypothesized mechanisms shown for this formulation were not directly evaluated in the trial. Overall, the findings were heterogeneous, short-term, and intervention-specific and should not be generalized across differently prepared or chemically characterized herbal products. The methodological characteristics of the eight included randomized controlled trials are presented [12,13,14,15,16,17,18,19].

**Table 1:** Search strategies applied in each bibliographic database and clinical trial registry.

Source	Search Strategy	Records Identified
PubMed/MEDLINE	(“Neoplasms”[Mesh] OR cancer[Title/Abstract] OR neoplasm*[Title/Abstract] OR tumor*[Title/Abstract] OR oncology[Title/Abstract]) AND (“Phytotherapy”[Mesh] OR phytotherapy[Title/Abstract] OR “herbal tea”[Title/Abstract] OR “herbal infusion”[Title/Abstract] OR “plant extract”[Title/Abstract] OR “herbal extract”[Title/Abstract] OR “leaf extract”[Title/Abstract] OR “botanical extract”[Title/Abstract] OR “medicinal plant*”[Title/Abstract] OR phytochemical*[Title/Abstract]) AND (“Clinical Trial”[Publication Type] OR “Randomized Controlled Trial”[Publication Type] OR “clinical trial”[Title/Abstract] OR “clinical study”[Title/Abstract] OR randomized[Title/Abstract] OR placebo[Title/Abstract] OR intervention[Title/Abstract])	1608
ScienceDirect	(herbal OR infusion OR tea) AND (cancer OR neoplasm) AND (randomized OR trial)	3984
Springer Nature Link	(“herbal infusion” OR “herbal tea” OR phytotherapy) AND (“clinical trial” OR “clinical study”)	696
CENTRAL	(herbal OR infusion OR tea) AND (cancer OR neoplasm) AND (randomized OR trial); and (“herbal infusion” OR “herbal tea” OR phytotherapy) AND (“clinical trial” OR “clinical study”)	119
Scopus	TITLE-ABS-KEY((cancer OR neoplasm* OR tumor* OR oncology) AND (phytotherapy OR “herbal tea” OR “herbal infusion” OR “plant extract” OR “herbal extract” OR “medicinal plant*” OR phytochemical*) AND (“clinical trial” OR “clinical study” OR randomized OR placebo OR intervention))	178
LILACS	(herbal OR infusion OR tea) AND (cancer OR neoplasm) AND (randomized OR trial); and (“herbal infusion” OR “herbal tea” OR phytotherapy) AND (“clinical trial” OR “clinical study”)	222
ClinicalTrials.gov	(herbal OR infusion OR tea) AND (cancer OR neoplasm) AND (randomized OR trial); and (“herbal infusion” OR “herbal tea” OR phytotherapy) AND (“clinical trial” OR “clinical study”)	640
WHO ICTRP	(herbal OR infusion OR tea) AND (cancer OR neoplasm) AND (randomized OR trial); and (“herbal infusion” OR “herbal tea” OR phytotherapy) AND (“clinical trial” OR “clinical study”)	28
Total		7475

Searches were conducted from database inception within the prespecified publication period of 1 January 1990, through 8 September 2026. Search syntax was adapted to the indexing system and technical functionality of each source. Counts represent records retrieved before deduplication. Embase and Web of Science were considered but could not be searched because institutional access was unavailable. We confirm that an explanation has been added to the table footer: * indicates the truncation operator used to retrieve different word endings and variants (e.g., neoplasm, neoplasms; tumor, tumors).

**Table 2:** Descriptive botanical and pharmaceutical characteristics of the plant-derived interventions reported in the 35 published reports with sufficient information for characterization.

Characteristic	Category	Reports (*n*)	Percentage (%)	Interpretation
Plant part used	Other	10	28.6	Botanical organ not specified or could not be classified.
	Mixed plant parts	8	22.9	Predominantly traditional multi-herb formulations (Kampo/TCM).
	Roots/rhizomes	5	14.3	Frequently associated with Asian medicinal plants (e.g., Panax, Isatis, Platycodon).
	Leaves	3	8.6	Mainly single-species preparations such as *Annona muricata* and *Camellia sinensis*.
	Flowers	3	8.6	Mainly represented by *Malva sylvestris* and *Alcea digitata*.
	Leaves/aerial parts	2	5.7	Including *Ocimum tenuiflorum* and *Cistus preparations*.
	Bark	1	2.9	Represented by *Handroanthus impetiginosus*.
	Seeds	1	2.9	Represented by *Paullinia cupana*.
	Flowers + leaves	1	2.9	Chamomile–peppermint formulation.
	Whole plant/aerial herb	1	2.9	*Scutellaria barbata*.
Most frequently reported botanical taxa	*Malva sylvestris*	3	8.6	Frequently investigated for radiation-induced xerostomia.
	*Alcea digitata*	3	8.6	Usually evaluated together with *Malva sylvestris*.
	*Panax ginseng*	4	11.4	Mainly included in Kampo formulations and fatigue-related studies.
	*Camellia sinensis*	3	8.6	Green tea preparations.
	*Panax quinquefolius*	2	5.7	Cancer-related fatigue interventions.
	*Viscum album*	2	5.7	Mistletoe supportive therapy.
	*Zingiber officinale*	2	5.7	Component of Kampo formulations.
	Other species	22	64.9	Each represented in only one study or reported within complex botanical formulations.
Pharmaceutical formulation	Granules/powder sachets	7	20.0	Mainly Kampo or traditional formulations.
	Herbal tea/infusion	5	14.3	Oral liquid preparations.
	Oral extract	5	14.3	Oral botanical extracts; the degree of chemical standardization varied or was not reported.
	Mouthwash/topical oral preparation	5	14.3	Primarily for oral mucositis.
	Other formulations	4	11.4	Preparations insufficiently described or not fitting the predefined categories.
	Capsules/solid oral products	3	8.6	Mostly ginseng preparations.
	Injectable extract	2	5.7	Mainly mistletoe therapy.
	Other multi-ingredient preparations	2	5.7	Complex botanical products.
	Dietary beverages	1	2.9	Beverage-based intervention.
	Other oral liquid preparation	1	2.9	Nutritional botanical preparation.
Methodological outcome	Included in qualitative synthesis	8	22.9	Fulfilled all predefined clinical eligibility criteria and were included in the qualitative synthesis.
	Published reports not included in the qualitative synthesis	27	77.1	Excluded from the qualitative synthesis but considered only in the descriptive characterization of full-text research trends.

Botanical and pharmaceutical characteristics of the plant-derived interventions reported in the 35 published reports with sufficient information for characterization. The two trial-registry records without associated publications reporting clinical results were not included in this descriptive analysis. Eight randomized controlled trials fulfilled the prespecified clinical eligibility criteria and were included in the qualitative synthesis, whereas 27 published reports were excluded from the synthesis. Twenty-eight of the 35 reports (80.0%) had descriptive Kitchenham-based scores of ≥9 points; however, this value was not considered a pass threshold, an eligibility criterion, or a risk-of-bias judgment. Kitchenham-based scores are reported exclusively to describe methodological and reporting characteristics. The frequencies presented in this table describe botanical and pharmaceutical research trends and should not be interpreted as evidence of clinical efficacy or safety. Species frequencies are non-mutually exclusive because several traditional formulations contain multiple medicinal plants.

**Table 3:** Clinical characteristics of the studies included in the qualitative synthesis.

Study	Country	Cancer Type	Clinical Stage	Intervention	Route	Duration	Main Clinical Outcome
Lee et al., 2018 [12]	Korea	Gastrointestinal, breast, and lung cancers	II–IV	Gamiguibi-tang (GGBT)	Oral	2 weeks	Improved sleep quality and reduced cancer-related fatigue
Yoshiya et al., 2020 [13]	Japan	Lung cancer	Pre-/postoperative and advanced	Rikkunshito	Oral	14 days	Improved chemotherapy-induced anorexia
Nomura et al., 2023 [14]	Japan	Differentiated thyroid cancer	I–IV	Holy basil (*Ocimum tenuiflorum*) tea	Oral	4 days	Reduced anxiety and preserved salivary function
Heydarirad et al., 2017 [15]	Iran	Head and neck cancer	Not reported	*Alcea digitata* + *Malva sylvestris* infusion	Oral	4 weeks	Improved xerostomia and quality of life
Indrawati et al., 2017 [16]	Indonesia	Colorectal cancer	I–IV	*Annona muricata* aqueous-leaf-extract-derived preparation administered in capsules	Oral	8 weeks	Increased cytotoxic activity and maintained nutritional status
Moeini et al., 2018 [17]	Iran	Prostate cancer	Not reported	*Malva sylvestris* L. flowers + *Althaea digitata* Boiss.	Oral	5 weeks	Reduced anal burning and diarrhea
Li et al., 2025 [18]	Taiwan	Gynecologic cancer	Not reported	Jing Si herbal tea (JSHT), 15 mL twice daily	Oral	6 weeks	No statistically significant between-group improvement in cancer-related fatigue or quality of life
Xu et al., 2020 [19]	China	Cancer survivors with different cancer diagnoses	Not reported	Renshen Yangrong Tang (RSYRT), a 12-herb extract-granule formulation, administered twice daily	Oral	6 weeks	Statistically and clinically greater reduction in fatigue severity and improvement in functioning compared with low-dose huangqi control.

The table summarizes the principal characteristics of the eight eligible randomized clinical trials, including cancer type, disease stage, intervention, route of administration, treatment duration, and main clinical outcome. All interventions were administered orally. Xu et al. (2020) [19] evaluated Renshen Yangrong Tang against a low-dose huangqi active control. Abbreviations: GGBT, Gamiguibi-tang; JSHT, Jing Si herbal tea; RSYRT, Renshen Yangrong Tang.

**Table 4:** Main clinical outcomes and effect estimates reported in the eight included randomized clinical trials.

Study	Intervention and Outcome	Participants	Main Effect Estimate	Follow-Up	Clinical Interpretation
Lee et al., 2018 [12]	Gamiguibi-tang (GGBT): insomnia and cancer-related fatigue	30	ISI change: −5.5 ± 4.4 vs. +0.1 ± 1.1 (*p* < 0.001); BFI change: −0.8 ± 0.8 vs. 0.0 ± 0.3 (*p* = 0.002)	2 weeks	Short-term improvements were reported, but the clinical importance remains uncertain because MCIDs and 95% CIs were not reported.
Yoshiya et al., 2020 [13]	Rikkunshito: chemotherapy-induced anorexia and plasma acylated ghrelin	31	Reduction in caloric intake: 18% vs. 25% (*p* = 0.025); greater recovery of plasma acylated ghrelin (*p* = 0.025)	14 days	A modest attenuation of reduced caloric intake was observed; the clinical relevance of the ghrelin finding remains uncertain.
Nomura et al., 2023 [14]	Holy basil (*Ocimum tenuiflorum*) tea: anxiety and salivary-gland function	NR	Lower anxiety scores and better preservation of salivary-gland function than control (*p* < 0.05); effect sizes and 95% CIs NR	4 days	Preliminary short-term findings; the magnitude and clinical importance of the effects could not be established.
Heydarirad et al., 2017 [15]	*Alcea digitata*–*Malva sylvestris* infusion: xerostomia and quality of life	NR	Improvement in xerostomia-related and overall EORTC QLQ-H&N35 scores versus artificial saliva (*p* < 0.05); effect size and 95% CI NR	4 weeks	Symptomatic improvement was reported, but its magnitude and clinical importance remain uncertain.
Indrawati et al., 2017 [16]	*Annona muricata* leaf preparation: nutritional status and exploratory ex vivo cytotoxicity	30 randomized; 28 analyzed	No significant between-group difference in nutritional status; greater ex vivo cytotoxic activity in patient serum than with placebo; numerical effect estimate NR	8 weeks	Nutritional benefit was not demonstrated. The exploratory surrogate finding does not establish clinical antitumor efficacy.
Moeini et al., 2018 [17]	*Malva sylvestris*–*Althaea digitata* preparation: anal burning and diarrhea during radiotherapy	NR	Lower anal-burning VAS score versus placebo; between-group difference in RTOG grading was not statistically significant; complete effect estimates and 95% CIs NR	5 weeks	A possible subjective symptom benefit was observed, but evidence for preventing radiation proctitis remains inconclusive.
Li et al., 2025 [18]	Jing Si herbal tea (JSHT): cancer-related fatigue and quality of life	21 enrolled; 19 analyzed	No statistically significant between-group differences in BFI-T or FACT-G7 scores; effect estimates and 95% CIs NR	6 weeks	The trial did not demonstrate a specific between-group benefit; within-group changes alone do not establish efficacy.
Xu et al., 2020 [19]	Renshen Yangrong Tang (RSYRT): cancer-related fatigue severity and functioning	83 evaluable: RSYRT, *n* = 41; low-dose huangqi control, *n* = 42	Greater reduction in MDASI fatigue score with RSYRT than with control: time-by-group interaction estimate = −0.61 (SE = 0.10; *p* < 0.0001). A reduction of at least two points was reported in 90.2% versus 52.4% of participants. At week 4, the mean reductions were −2.66 versus −1.36, respectively (Cohen’s *d* = 1.0; *p* < 0.0001).	6 weeks	RSYRT produced a statistically significant and potentially clinically relevant short-term reduction in fatigue compared with a low-dose active herbal control. However, the modest sample size, short follow-up, and absence of 95% CIs limit the certainty and precision of the finding.

BFI, Brief Fatigue Inventory; BFI-T, Brief Fatigue Inventory–Taiwan Form; CI, confidence interval; EORTC QLQ-H&N35, European Organisation for Research and Treatment of Cancer Quality of Life Questionnaire–Head and Neck Module; FACT-G7, Functional Assessment of Cancer Therapy–General 7-item version; GGBT, *Gamiguibi-tang*; ISI, Insomnia Severity Index; JSHT, Jing Si herbal tea; MCID, minimal clinically important difference; MDASI, MD Anderson Symptom Inventory; NR, not reported; RSYRT, Renshen Yangrong Tang; RTOG, Radiation Therapy Oncology Group; SE, standard error; VAS, visual analog scale. Methodological and reporting characteristics are presented separately in Table 5, whereas detailed RoB 2 assessments are provided in Supplementary Table S4 and Supplementary Figure S2. Statistical significance should not be interpreted as equivalent to clinically meaningful or definitive efficacy.

**Table 5:** Methodological and reporting characteristics of the eight included randomized clinical trials.

Methodological Characteristic	Lee et al. [12]	Yoshiya et al. [13]	Nomura et al. [14]	Heydarirad et al. [15]	Indrawati et al. [16]	Moeini et al. [17]	Li et al., 2025 [18]	Xu et al., 2020 [19]
Randomization	Randomized parallel-group pilot trial; sequence-generation method reported	Randomized crossover trial; participants assigned to treatment sequences	Randomized parallel-group trial	Randomized active-controlled trial; sequence-generation details not reported	Block randomization, with four participants per block	Randomized multicenter trial; sequence-generation details not reported	Randomized two-group trial; sequence-generation method not clearly reported	Randomized parallel-group Phase II trial; sequence-generation method not reported
Allocation concealment	Concealed allocation reported	Not reported	Not reported	Not reported	Not reported	Not reported	Not reported	Not reported
Blinding	Open-label	Open-label	Participant and investigator blinding not reported	Open-label	Double-blind	Double-blind	Blinding procedures not clearly reported	Blinding procedures not clearly reported
Placebo/control	Wait-list control; no placebo	No-treatment crossover control; no placebo	Control group without placebo tea	Artificial-saliva active comparator; no placebo infusion	Maltose placebo capsule	Placebo infusion	Placebo-controlled delayed-treatment design; placebo for 3 weeks followed by JSHT for 3 weeks	Active herbal control consisting of low-dose huangqi; no inert placebo
Sample-size calculation	Pilot study; formal calculation not reported	Pilot study; formal calculation not reported	Reported	Not reported	Not reported	Not reported	Reported; planned enrollment was not achieved	Not reported
ITT analysis	Not reported	No; per-protocol analysis	Not reported	Not reported	No; 28 of 30 randomized participants completed the study and were analyzed	Not reported	No; 19 of 21 enrolled participants were analyzed	Not clearly reported; analysis was based on 83 evaluable participants
Attrition	Withdrawals reported	Participants were excluded after randomization	Participant flow reported	Attrition reported	Two participants withdrew	Withdrawals reported	Two enrolled participants were not analyzed; imputation method not reported	Number and reasons for post-randomization exclusions or withdrawals not clearly reported
Missing-data handling	Complete-case analysis used; imputation not reported	Not reported	Missing-data imputation not reported	Not reported	No imputation method reported	Not reported	Not reported	Not reported
Adherence	Assessed through returned medication and participant reporting; quantitative adherence threshold not clearly reported	Not reported	Tea consumption monitored during hospitalization; quantitative adherence analysis not reported	Not reported	Follow-up conducted every two weeks; quantitative adherence rate not reported	Not reported	Not reported	Not reported
Multiple-testing correction	Not reported	Not reported	Not reported	Not reported	Not reported	Not reported	Not reported	Not reported
Validated outcomes	Validated patient-reported instruments: ISI and BFI	No validated patient-reported instrument reported; caloric intake and plasma acylated ghrelin were assessed as objective endpoints	Validated patient-reported instrument: STAI; salivary-gland scintigraphy was used as an objective assessment	Validated patient-reported instrument: EORTC QLQ-H&N35; additional xerostomia-related assessments were reported	No validated patient-reported instrument reported; anthropometric and dietary assessments were used, and ex vivo cytotoxicity was an exploratory surrogate endpoint	RTOG grading and VAS symptom assessment	BFI-T and FACT-G7	Validated patient-reported outcome: MD Anderson Symptom Inventory fatigue score
Funding	Not reported	Not reported	Not reported	Not reported	Centre for Ageing Studies, Universitas Indonesia	Not reported	Not reported	Key Program Foundation of Beijing Administration of TCM; the funder had no role in the study design, conduct, analysis, interpretation, or reporting.
Chemical characterization	Standardized commercial GGBT formulation; trial-specific quantitative phytochemical profiling not reported	Standardized commercial Rikkunshito product; trial-specific chromatographic or quantitative phytochemical characterization not reported	Botanical preparation described; chromatographic fingerprinting and marker-compound quantification not reported	Botanical ingredients and preparation procedure described; quantitative phytochemical characterization not reported	Ethanol-soluble fraction of the aqueous leaf extract standardized to 0.36% acetogenins	Botanical authentication and preparation described; chromatographic fingerprinting and quantitative marker-compound analysis not reported	Eight-herb formulation described; trial-specific chromatographic fingerprinting and marker-compound quantification not reported	Twelve-herb RSYRT extract-granule formulation described; trial-specific chromatographic fingerprinting, validated marker-compound quantification, and batch-level chemical characterization not clearly reported

BFI, Brief Fatigue Inventory; EORTC QLQ-H&N35, European Organisation for Research and Treatment of Cancer Quality of Life Questionnaire–Head and Neck Module; FACT-G7, Functional Assessment of Cancer Therapy–General 7-item version; GGBT, Gamiguibi-tang; ISI, Insomnia Severity Index; ITT, intention-to-treat; JSHT, Jing Si herbal tea; RSYRT, Renshen Yangrong Tang; RTOG, Radiation Therapy Oncology Group; STAI, State–Trait Anxiety Inventory; VAS, visual analog scale. “Not reported” indicates that the corresponding information was not explicitly provided in the primary publication. The Kitchenham-based instrument was used descriptively and was not used as an eligibility threshold or as a substitute for the RoB 2 risk-of-bias assessment. Unless explicitly indicated, plant-to-water ratios, preparation temperature and duration, chromatographic fingerprints, validated marker compounds, batch-to-batch consistency, GMP manufacturing information, pharmacokinetics, bioavailability, and herb–drug interaction testing were not reported in the primary trial publications. Botanical authentication, a general product description, or the use of a commercial formulation was not considered equivalent to comprehensive trial-specific chemical standardization. The reported standardization of the *Annona muricata* preparation to 0.36% acetogenins represents limited marker-compound standardization and should not be interpreted as complete phytochemical or manufacturing characterization.

**Table 6:** Literature-derived phytochemical constituents and hypothesized pharmacological mechanisms, together with the clinical outcomes reported for the herbal preparations evaluated in the eight included randomized clinical trials.

Orally Administered Herbal Preparation	Cancer Type	Principal Phytochemicals	Proposed Pharmacological Mechanisms	Main Reported Clinical Outcome
Gamiguibi-tang (GGBT)	Gastrointestinal, breast, lung	Multiple flavonoids, triterpenoid saponins, polysaccharides	Anti-inflammatory, neuroprotective, immunomodulatory	Improved sleep quality and reduced fatigue
Rikkunshito	Lung	Ginsenosides, hesperidin, gingerols	Ghrelin modulation, gastrointestinal protection	Reduced chemotherapy-induced anorexia
Holy basil (*Ocimum tenuiflorum*)	Thyroid	Eugenol, rosmarinic acid, ursolic acid	Antioxidant, anxiolytic	Reduced anxiety and preserved salivary function
*Malva sylvestris* + *Alcea digitata*	Head and neck	Mucilage, flavonoids, phenolic compounds	Demulcent, anti-inflammatory	Improved xerostomia and quality of life
*Annona muricata*	Colorectal	Acetogenins, phenolic compounds	Proposed antiproliferative and cytotoxic activity	No significant between-group difference in nutritional status; greater exploratory ex vivo cytotoxic activity was reported
*Malva sylvestris* + *Althaea digitata*	Prostate	Mucilage, flavonoids	Anti-inflammatory, mucosal protection	Reduced anal burning and diarrhea
*Jing Si herbal tea* (JSHT)	Gynecologic cancer	Literature-derived flavonoids, phenolic compounds, polysaccharides, and saponins associated with its multi-herb composition	Hypothesized anti-inflammatory, immunomodulatory, and antioxidant activities	No statistically significant between-group differences in cancer-related fatigue or quality of life
*Renshen Yangrong Tang (RSYRT), a 12-herb extract-granule formulation*	Cancer survivors with different cancer diagnoses	Literature-derived ginsenosides, astragalosides, polysaccharides, and flavonoids associated with its multi-herb composition	Hypothesized immunomodulatory, anti-inflammatory, neuroendocrine, and fatigue-modulating activities	Greater short-term reduction in cancer-related fatigue severity and improvement in functioning compared with low-dose huangqi control

The representative phytochemicals and hypothesized pharmacological mechanisms summarized in this table were derived from the broader phytochemical, pharmacological, and preclinical literature. They were not directly identified, quantified, or mechanistically evaluated in the eight included RCTs and should not be interpreted as demonstrated explanations for the reported clinical outcomes. Trial-specific chromatographic fingerprinting and quantitative marker-compound analyses were generally unavailable. Clinically relevant systemic exposure was also not established because the included trials did not adequately evaluate aqueous-extraction efficiency, bioavailability, absorption, metabolism, pharmacokinetics, circulating concentrations, or herb–drug interactions involving the proposed constituents or their metabolites. The clinical findings should therefore be interpreted as preparation-specific and should not be generalized across chemically heterogeneous or differently standardized herbal products. Abbreviations: GGBT, Gamiguibi-tang; JSHT, Jing Si herbal tea; RSYRT, Renshen Yangrong Tang.

## Data Availability

No new data were created or analyzed in this study. Data sharing is not applicable to this article.

## Supplementary Materials

The following supporting information can be downloaded at: https://www.mdpi.com/article/10.3390/medsci14060597/s1, Table S1. Descriptive methodological and reporting assessment of the 35 published reports with sufficient information for evaluation. Table S2. Reports and trial-registry records excluded after eligibility assessment, with one primary reason for exclusion assigned to each record (*n* = 29). Table S3. GRADE Summary of Findings—Outcome-level certainty-of-evidence assessment for the eight included randomized controlled trials. Table S4. Domain-level risk-of-bias assessment of the principal outcomes of the eight included randomized controlled trials according to the Cochrane Risk of Bias 2 (RoB 2) tool. Figure S1. Intervention-by-outcome evidence-gap heatmap for the eight included randomized controlled trials. Dark-blue cells containing an upward arrow (↑) indicate a statistically significant favorable direction of effect; light-blue cells containing a horizontal arrow (↔) indicate no clear between-group benefit or maintenance without demonstrated superiority; cells containing both symbols (↑/↔) indicate mixed findings within the same outcome domain; and light-gray cells containing a dash (—) indicate that the outcome was not evaluated. Empty evidence domains should not be interpreted as evidence of no effect. The superscript number indicates the number of RCTs contributing evidence to each intervention–outcome relationship (^1^ = one RCT). All evaluated relationships were supported by a single trial and were rated as very-low-certainty evidence. The final column presents the overall RoB 2 judgment: yellow indicates some concerns and red indicates high risk of bias. Abbreviations: RCT, randomized controlled trial; RoB 2, revised Cochrane Risk of Bias tool for randomized trials; RSYRT, Renshen Yangrong Tang; RTOG, Radiation Therapy Oncology Group. Figure S2. Risk-of-bias assessment of the principal outcomes of the eight included randomized controlled trials according to the revised Cochrane Risk of Bias tool for randomized trials (RoB 2). Green circles indicate low risk of bias, yellow circles indicate some concerns, and red circles indicate high risk of bias. Overall judgments were derived from the domain-level assessments and were not based on the descriptive Kitchenham instrument. Abbreviation: RoB 2, revised Cochrane Risk of Bias tool for randomized trials. Figure S3. Translational roadmap for the clinical development of orally administered herbal preparations in supportive cancer care. The proposed framework emphasizes sequential botanical authentication, chemical standardization, pharmacokinetic and bioavailability assessment, herb–drug interaction and safety testing, adequately powered multicenter randomized controlled trials, and long-term pharmacovigilance. Each stage addresses evidence gaps identified across the included trials and should be completed before broader clinical implementation. Abbreviations: ITT, intention-to-treat; MCID, minimal clinically important difference; RCT, randomized controlled trial. The eight included randomized controlled trials underlying Tables S1, S3 and S4 and Figures S1 and S2 are cited in the main reference list [12,13,14,15,16,17,18,19]. The reports and trial-registry records evaluated or excluded during full-text assessment and presented in Tables S1 and S2 are also included in the main reference list [47,48,49,50,51,52,53,54,55,56,57,58,59,60,61,62,63,64,65,66,67,68,69,70,71,72,73,74,75].

## Abbreviations

The following abbreviations are used in this manuscript:

PROSPERO	Prospective Register Of Systematic Reviews Only
PICOC	Population, Intervention, Comparison, Outcomes, and Context
GGBT	Gamiguibi-tang
RoB 2	Risk of Bias 2
QCD	Quality of Clinical Desing
QCC	Quality of Clinical Conduct
QCA	Quality of Clinical Analysis
QCCo	Quality of Clinical Conclusions
