# Supplementary material for: Clinical Evidence of Orally Administered Herbal Preparations for Supportive Cancer Care: A PRISMA Systematic Review of Randomized Clinical Trials (1990–2026)
Source: Med Sci (Basel). 2026 Sep 22;14(6):597. doi: 10.3390/medsci14060597 (PMC13706724; doi:10.3390/medsci14060597)
Supplement: Supplementary file 1 [file medsci-14-00597-s001.zip › medsci-4511279-PRISMA checklist.pdf]

PRISMA 2020 Checklist

Section and Topic	Item #	Checklist item	Location where item is reported
**TITLE**			
Title	1	Identify the report as a systematic review.	Title page (manuscript title).
**ABSTRACT**			
Abstract	2	See the PRISMA 2020 for Abstracts checklist.	Abstract (structured Background/Objectives, Methods, Results, and Conclusions).
**INTRODUCTION**			
Rationale	3	Describe the rationale for the review in the context of existing knowledge.	Section 1, Introduction (rationale and context of the available evidence).
Objectives	4	Provide an explicit statement of the objective(s) or question(s) the review addresses.	Section 1, Introduction (final paragraph: review objective).
**METHODS**			
Eligibility criteria	5	Specify the inclusion and exclusion criteria for the review and how studies were grouped for the syntheses.	Sections 2.4 and 3.7; studies were grouped and interpreted by herbal intervention, cancer population/treatment context, and outcome domain.
Information sources	6	Specify all databases, registers, websites, organisations, reference lists and other sources searched or consulted to identify studies. Specify the date when each source was last searched or consulted.	Section 2.2 and Table 1 (databases, trial registries, search dates, and coverage period).
Search strategy	7	Present the full search strategies for all databases, registers and websites, including any filters and limits used.	Table 1 and Supplementary Materials (complete database- and registry-specific search strategies, dates, and filters where applicable).
Selection process	8	Specify the methods used to decide whether a study met the inclusion criteria of the review, including how many reviewers screened each record and each report retrieved, whether they worked independently, and if applicable, details of automation tools used in the process.	Section 2.3 (two independent reviewers, sequential title/abstract and full-text screening, consensus and third-reviewer procedures).
Data collection process	9	Specify the methods used to collect data from reports, including how many reviewers collected data from each report, whether they worked independently, any processes for obtaining or confirming data from study investigators, and if applicable, details of automation tools used in the process.	Section 2.5 (independent duplicate extraction using a standardized form; disagreements resolved by joint re-examination and consensus).
Data items	10a	List and define all outcomes for which data were sought. Specify whether all results that were compatible with each outcome domain in each study were sought (e.g. for all measures, time points, analyses), and if not, the methods used to decide which results to collect.	Section 2.5 and Table 4 (efficacy and safety outcomes and extracted effect information).
	10b	List and define all other variables for which data were sought (e.g. participant and intervention characteristics, funding sources). Describe any assumptions made about any missing or unclear information.	Section 2.5 and Tables 2-3 (bibliographic, population, cancer, intervention, comparator, route, dose, duration, and formulation variables).
Study risk of bias assessment	11	Specify the methods used to assess risk of bias in the included studies, including details of the tool(s) used, how many reviewers assessed each study and whether they worked independently, and if applicable, details of automation tools used in the process.	Section 2.6 (Kitchenham-based descriptive assessment and outcome-level Cochrane RoB 2 assessment by two independent reviewers).
Effect measures	12	Specify for each outcome the effect measure(s) (e.g. risk ratio, mean difference) used in the synthesis or presentation of results.	Section 2.5 and Table 4. Effect estimates were reported as presented by each trial, including between-group mean differences, changes from baseline, relative effect measures, confidence intervals, and p-values when available. No common summary measure was calculated.
Synthesis methods	13a	Describe the processes used to decide which studies were eligible for each synthesis (e.g. tabulating the study intervention characteristics and comparing against the planned groups for each synthesis (item #5)).	Sections 2.4, 2.6, and 3.7 (eligibility for qualitative synthesis and grouping by intervention-outcome relationship).
	13b	Describe any methods required to prepare the data for presentation or synthesis, such as handling of missing summary statistics, or data conversions.	Section 2.5. No statistical conversion or imputation was undertaken; effect estimates were reported as presented in the original trials, with unreported information identified as NR.
	13c	Describe any methods used to tabulate or visually display results of individual studies and syntheses.	Tables 2-6, Figure 2, Supplementary Tables S1-S4, and Supplementary Figures S1-S3.
	13d	Describe any methods used to synthesize results and provide a rationale for the choice(s). If meta-analysis was performed, describe the model(s), method(s) to identify the presence and extent of statistical heterogeneity, and software	Sections 2.1 and 3.7. Results were synthesized narratively; meta-analysis was not undertaken because

PRISMA 2020 Checklist

Section and Topic	Item #	Checklist item	Location where item is reported
		package(s) used.	clinically comparable intervention-outcome groups could not be established.
	13e	Describe any methods used to explore possible causes of heterogeneity among study results (e.g. subgroup analysis, meta-regression).	Section 3.7 and Section 4. Sources of clinical and methodological heterogeneity were described narratively; no statistical subgroup or meta-regression analysis was performed.
	13f	Describe any sensitivity analyses conducted to assess robustness of the synthesized results.	Not applicable. No quantitative synthesis or meta-analysis was performed; therefore, no sensitivity analysis of a pooled estimate was possible.
Reporting bias assessment	14	Describe any methods used to assess risk of bias due to missing results in a synthesis (arising from reporting biases).	Section 2.4. Formal assessment of reporting bias or small-study effects was not feasible because no meta-analysis or comparable synthesis containing multiple studies was available.
Certainty assessment	15	Describe any methods used to assess certainty (or confidence) in the body of evidence for an outcome.	Section 2.6. Outcome-level certainty was assessed using GRADE independently from RoB 2 and the descriptive methodological assessment.
**RESULTS**			
Study selection	16a	Describe the results of the search and selection process, from the number of records identified in the search to the number of studies included in the review, ideally using a flow diagram.	Section 3.1 and Figure 1 (PRISMA 2020 flow diagram).
	16b	Cite studies that might appear to meet the inclusion criteria, but which were excluded, and explain why they were excluded.	Section 3.1 and Supplementary Table S2 (29 excluded reports/registry records, each with one primary exclusion reason).
Study characteristics	17	Cite each included study and present its characteristics.	Section 3.3 and Table 3 (characteristics of the eight included randomized controlled trials).
Risk of bias in studies	18	Present assessments of risk of bias for each included study.	Section 3.8, Table 5, Supplementary Table S4, and Supplementary Figure S2.
Results of individual studies	19	For all outcomes, present, for each study: (a) summary statistics for each group (where appropriate) and (b) an effect estimate and its precision (e.g. confidence/credible interval), ideally using structured tables or plots.	Section 3.5 and Table 4 (study-level outcomes and effect estimates, including confidence intervals and p-values when reported).
Results of syntheses	20a	For each synthesis, briefly summarise the characteristics and risk of bias among contributing studies.	Sections 3.7-3.8, Table 5, Supplementary Table S4, and Supplementary Figure S2.
	20b	Present results of all statistical syntheses conducted. If meta-analysis was done, present for each the summary estimate and its precision (e.g. confidence/credible interval) and measures of statistical heterogeneity. If comparing groups, describe the direction of the effect.	Section 3.7. No statistical synthesis or meta-analysis was conducted; trial-level findings were synthesized narratively.
	20c	Present results of all investigations of possible causes of heterogeneity among study results.	Section 3.7 and Section 4. Heterogeneity was considered narratively; no formal statistical investigation was possible because comparable intervention-outcome groups were unavailable.
	20d	Present results of all sensitivity analyses conducted to assess the robustness of the synthesized results.	Not applicable. No quantitative synthesis was conducted and therefore no sensitivity analysis was performed.
Reporting biases	21	Present assessments of risk of bias due to missing results (arising from reporting biases) for each synthesis assessed.	Section 2.4. A formal reporting-bias assessment was not feasible because each intervention-outcome relationship was generally represented by a single trial and no meta-analysis was performed.
Certainty of evidence	22	Present assessments of certainty (or confidence) in the body of evidence for each outcome assessed.	Section 3.7 and Supplementary Table S3 (outcome-level GRADE assessments; all evaluated outcomes rated very low certainty).
**DISCUSSION**			
Discussion	23a	Provide a general interpretation of the results in the context of other evidence.	Section 4, Discussion (interpretation in relation to prior evidence and evidentiary standards).
	23b	Discuss any limitations of the evidence included in the review.	Section 4, Discussion (risk of bias, imprecision, small samples, heterogeneity, botanical/chemical

PRISMA

PRISMA 2020 Checklist

Section and Topic	Item #	Checklist item	Location where item is reported
			standardization, and safety limitations).
	23c	Discuss any limitations of the review processes used.	Section 4, Discussion (limitations of database access, English-language restriction, search completeness, and inability to perform quantitative synthesis).
	23d	Discuss implications of the results for practice, policy, and future research.	Section 4, Section 5 Conclusions, and Supplementary Figure S3 (intervention-specific interpretation and research implications).
**OTHER INFORMATION**			
Registration and protocol	24a	Provide registration information for the review, including register name and registration number, or state that the review was not registered.	Section 2.1 (PROSPERO registration CRD420261438444).
	24b	Indicate where the review protocol can be accessed, or state that a protocol was not prepared.	Section 2.1; the prospectively registered review record is available in PROSPERO under CRD420261438444.
	24c	Describe and explain any amendments to information provided at registration or in the protocol.	Section 2.1. The database and registry search was expanded in response to peer review, and the amendment is reported transparently.
Support	25	Describe sources of financial or non-financial support for the review, and the role of the funders or sponsors in the review.	Funding statement.
Competing interests	26	Declare any competing interests of review authors.	Conflicts of Interest statement.
Availability of data, code and other materials	27	Report which of the following are publicly available and where they can be found: template data collection forms; data extracted from included studies; data used for all analyses; analytic code; any other materials used in the review.	Data Availability Statement and Supplementary Materials statement (Tables S1-S4 and Figures S1-S3).

From: Page MJ, McKenzie JE, Bossuyt PM, Boutron I, Hoffmann TC, Mulrow CD, et al. The PRISMA 2020 statement: an updated guideline for reporting systematic reviews. BMJ 2021;372:n71. doi: 10.1136/bmj.n71. This work is licensed under CC BY 4.0. To view a copy of this license, visit https://creativecommons.org/licenses/by/4.0/
