# Supplementary material for: Clinical Evidence of Orally Administered Herbal Preparations for Supportive Cancer Care: A PRISMA Systematic Review of Randomized Clinical Trials (1990–2026)
Source: Med Sci (Basel). 2026 Sep 22;14(6):597. doi: 10.3390/medsci14060597 (PMC13706724; doi:10.3390/medsci14060597)
Supplement: Supplementary file 1 [file medsci-14-00597-s001.zip › medsci-4511279-supplementary.pdf]

medical sciences

MDPI

Supplementary Material

Table S1. Descriptive methodological and reporting assessment of the 35 published reports with sufficient information for evaluation.

No.	Study	Q1	Q2	Q3	Q4	Q5	Q6	Q7	Q8	Q9	Q10	Q11	Total	Clinical eligibility
1	Wisconsin ginseng for cancer-related fatigue	1	0	1	1	0	1	1	1	0	1	5	12	Excluded
2	*Uncaria tomentosa* and quality of life in advanced solid tumors	1	0	1	0	0	1	1	0	1	0	5	10	Excluded
3	Daikenchuto after total gastrectomy for gastric cancer	0	1	1	1	0	1	1	0	1	0	5	11	Excluded
4	Adjuvant mistletoe treatment in resected head and neck cancer	0	1	1	1	1	1	1	P	0	0	5	11	Excluded
5	Herbal agent MB-6 with chemotherapy in colorectal cancer	0	1	1	1	0	1	1	1	0	1	5	12	Excluded
6	Traditional herbal medicine after hepatocellular carcinoma resection	1	1	1	1	1	1	1	1	1	0	0	9	Excluded
7	Moeini et al., 2018—*Malva sylvestris–Althaea digitata*	1	1	1	0	1	1	1	P	1	1	5	13	**Included**
8	Li et al., 2025—Jing Si herbal tea for fatigue in gynecologic cancer<br>Clinical eligibility: Included	0	1	P	1	1	1	1	1	1	0	5	12	**Included**
9	Indrawati et al., 2017—*Annona muricata* preparation	NR	NR	NR	NR	NR	NR	NR	NR	NR	NR	5	5	**Included**
10	Cystus tea mouthwash for radiation-induced mucositis	1	1	1	0	1	1	1	1	1	1	5	14	Excluded
11	Herbal preparation with glycerin for radiation-induced oral mucositis	1	1	1	0	1	1	1	1	1	0	0	8	Excluded
12	Yoshiya et al., 2020—Rikkunshito for chemotherapy-induced anorexia	1	1	1	0	1	1	1	1	1	1	5	14	**Included**

13	Phase I trial of oral green-tea extract in solid tumors	1	0	0	0	0	0	0	0	0	1	5	7	Excluded
14	Oral Lapacho-based medication for supportive care	0	0	1	0	0	1	0	0	0	0	5	6	Excluded
15	Chemotherapy with or without traditional Chinese medicine in lung cancer	1	1	1	1	0	0	1	0	0	0	5	10	Excluded
16	Molecular mistletoe therapy in pancreatic cancer	1	1	0	1	0	1	1	1	0	1	5	12	Excluded
17	Ninjin'Yoeito for fatigue during pancreatic-cancer treatment	1	1	1	0	0	1	1	0	0	0	5	9	Excluded
18	Guarana for anorexia in advanced cancer	1	1	1	1	0	1	1	0	1	0	5	12	Excluded
19	Herbal compound for dry mouth in head and neck cancer	1	1	1	0	0	1	1	0	0	0	5	9	Excluded
20	*Matricaria recutita–Mentha piperita* herbal mouthwash	1	1	1	0	0	1	1	0	0	0	5	9	Excluded
21	Heydarirad et al., 2017—*Alcea digitata–Malva sylvestris*	1	1	1	0	1	1	1	1	1	1	5	14	**Included**
22	Lee et al., 2018—Gamiguibi-tang for cancer-related sleep disturbance	1	1	1	1	1	1	1	1	1	1	5	14	**Included**
23	Sun ginseng and quality of life in patients with cancer	0	1	1	0	0	1	0	0	0	0	3	6	Excluded
24	*Isatis indigotica* for radiation-induced mucositis	1	1	1	0	0	1	1	1	0	0	3	9	Excluded
25	Natural-agent mixture for prevention of acute mucositis	1	1	1	0	0	1	1	1	1	1	2	9	Excluded
26	Antioxidant-rich beverages and breast-cancer risk	1	0	0	1	0	1	1	1	1	1	2	8	Excluded
27	Herbal medicine for cancer-induced anorexia/cachexia	1	1	1	0	0	1	1	1	1	0	5	12	Excluded
28	Chinese herbal medicine as maintenance therapy in lung cancer	1	1	1	0	0	1	1	1	1	0	5	12	Excluded

29	*Platycodon grandiflorum* in breast cancer: study protocol	1	1	1	1	0	1	1	0	0	0	5	10	Excluded
30	Nomura et al., 2023—Holy basil tea during radioiodine therapy	1	1	1	1	0	1	1	0	1	1	5	13	**Included**
31	Adjunctive traditional Chinese medicine in advanced lung cancer	1	1	1	1	0	1	1	0	1	0	5	11	Excluded
32	Ginseng for fatigue in head and neck cancer	1	1	1	0	0	1	1	0	1	1	5	12	Excluded
33	Phase II trial of green tea in metastatic prostate cancer	1	1	1	0	0	0	0	P	0	0	5	8	Excluded
34	Phase 1B trial of *Scutellaria barbata* in metastatic breast cancer	0	0	1	1	1	1	0	0	0	0	5	9	Excluded
35	Xu et al., 2020—Renshen Yangrong Tang for cancer-related fatigue in cancer survivors	1	0	1	1	1	1	1	1	1	1	5	14	**Included**

Assessment criteria: Q1, clearly stated objective or hypothesis; Q2, appropriate control group and description of cancer stage; Q3, clearly described participant eligibility criteria; Q4, safety monitoring; Q5, appropriate definition and measurement of efficacy outcomes; Q6, ethics approval and/or informed consent; Q7, clearly described statistical methods; Q8, adequate analysis of clinically relevant safety and efficacy outcomes; Q9, conclusions supported by the reported results; Q10, acknowledgment of limitations or potential biases; and Q11, adequate identification of the herbal intervention and correspondence with the predefined oral-administration and formulation criteria. Scoring: Q1–Q10, 1 when the criterion was fulfilled and 0 when it was not fulfilled or not reported; Q11, 5 when the intervention corresponded to the predefined botanical, formulation, and oral-administration criteria, with lower values indicating incomplete correspondence. NR, not reported or not assessable from the available information. The maximum descriptive score was 15 points. Scores were used exclusively to characterize methodological and reporting features and were not used as an eligibility threshold, risk-of-bias judgment, or substitute for RoB 2. Final eligibility was determined independently according to the prespecified population, intervention, formulation, route, outcome, and study-design criteria.

Table S2. Reports and trial-registry records excluded after eligibility assessment, with one primary reason for exclusion assigned to each record (n = 29).

No.	Authors, year	Title	Journal	Primary reason for exclusion
1	Barton et al., 2013	Wisconsin ginseng (*Panax quinquefolius*) to improve cancer-related fatigue: A randomized, double-blind trial, N07C2	*Journal of the National Cancer Institute*	**Ineligible formulation:** The intervention consisted of encapsulated powdered American ginseng root rather than a preparation documented as originating from an infusion, decoction, or eligible aqueous-extraction process. The capsule

				dosage form was not itself the reason for exclusion.
2	de Paula et al., 2015	*Uncaria tomentosa* (cat's claw) improves quality of life in patients with advanced solid tumors	*Journal of Alternative and Complementary Medicine*	**Ineligible study design:** Prospective single-arm Phase II study without an eligible randomized comparator.
3	Yoshikawa et al., 2015	Effect of Daikenchuto, a traditional Japanese herbal medicine, after total gastrectomy for gastric cancer: A multicenter, randomized, double-blind, placebo-controlled Phase II trial	*Journal of the American College of Surgeons*	**Insufficient formulation information:** The report described Daikenchuto as pharmaceutical-grade extract granules but did not document the extraction solvent or provide sufficient evidence that the administered product originated from an infusion, decoction, or eligible aqueous-extraction process. The final granule dosage form was not itself the reason for exclusion.
4	Steuer-Vogt et al., 2001	The effect of an adjuvant mistletoe treatment programme in resected head and neck cancer patients: A randomised controlled clinical trial	*European Journal of Cancer*	**Ineligible route:** The mistletoe extract was administered subcutaneously rather than through an eligible orally ingested route.
5	Chen et al., 2014	Effectiveness of a novel herbal agent MB-6 as a potential adjunct to 5-fluorouracil-based chemotherapy in colorectal cancer	*Nutrition Research*	**Ineligible intervention:** MB-6 was a proprietary multi-ingredient product for which correspondence with the prespecified infusion-, decoction-, or eligible aqueous-extract-derived intervention definition could not be established.
6	Yue et al., 2025	Traditional herbal medicine as an adjuvant therapy for preventing the recurrence of hepatocellular carcinoma after radical resection: Development and validation of a machine-learning prediction model	*European Journal of Integrative Medicine*	**Ineligible study design:** The report presented the development and validation of a prediction model and did not report an eligible randomized clinical trial.
7	Ebert et al., 2021	Results of a randomized controlled Phase III trial: Efficacy of polyphenol-containing Cystus tea mouthwash solution for the	*Strahlentherapie und Onkologie*	**Ineligible route:** The herbal preparation was used as a topical mouthwash and was not ingested.

		reduction of mucositis in head and neck cancer patients undergoing external beam radiotherapy		
8	Putwatana et al., 2009	Relief of radiation-induced oral mucositis in head and neck cancer	*Cancer Nursing*	**Ineligible route:** The glycerin-based herbal preparation was applied topically inside the oral cavity rather than ingested.
9	Pisters et al., 2001	Phase I trial of oral green tea extract in adult patients with solid tumors	*Journal of Clinical Oncology*	**Ineligible study design:** Phase I dose-escalation study without an eligible randomized comparator. The solid dosage form was not used as the reason for exclusion.
10	Giacomelli et al., 2015	Oral Lapacho-based medication: An easy, safe, and feasible support to prevent and/or reduce oral mucositis during radiotherapy for head and neck cancer	*Nutrition and Cancer*	**Ineligible study design:** Non-randomized Phase II study without an eligible randomized comparator.
11	Xu et al., 2012	Multi-center randomized double-blind controlled clinical study of chemotherapy combined with or without traditional Chinese medicine on quality of life of postoperative non-small-cell lung cancer patients	*BMC Complementary and Alternative Medicine*	**Insufficient formulation information:** The report did not provide sufficient information on the preparation method or extraction solvent to establish that the administered intervention originated from an infusion, decoction, or eligible aqueous-extraction process.
12	Matthes et al., 2010	Molecular mistletoe therapy: Friend or foe in established anti-tumor protocols? A multicenter, controlled, retrospective pharmaco-epidemiological study in pancreatic cancer	*Current Molecular Medicine*	**Ineligible study design:** Retrospective, non-randomized pharmaco-epidemiological study.
13	Okada et al., 2020	Impact of Ninjin'Yoeito on fatigue in patients receiving nab-paclitaxel plus gemcitabine therapy: A prospective, single-arm, Phase II open-label, nonrandomized, historically controlled study	*Current Therapeutic Research*	**Ineligible study design:** Prospective, single-arm, non-randomized Phase II study with a historical comparison.

14	Palma et al., 2016	Guarana (*Paullinia cupana*) improves anorexia in patients with advanced cancer	*Journal of Dietary Supplements*	**Ineligible study design:** Prospective single-arm study without an eligible randomized comparator. The dry-extract presentation was not used as the reason for exclusion.
15	Ameri et al., 2016	Evaluation of efficacy of an herbal compound on dry mouth in patients with head and neck cancers: A randomized clinical trial	*Journal of Evidence-Based Complementary and Alternative Medicine*	**Insufficient formulation information:** The report described a powder-based herbal compound but did not provide sufficient evidence that it originated from an infusion, decoction, or eligible aqueous-extraction process.
16	Tavakoli Ardakani et al., 2016	Evaluating the effect of *Matricaria recutita* and *Mentha piperita* herbal mouthwash on the management of oral mucositis in patients undergoing hematopoietic stem-cell transplantation: A randomized, double-blind, placebo-controlled clinical trial	*Complementary Therapies in Medicine*	**Ineligible route:** The intervention was administered as a topical mouthwash and was not ingested.
17	Kim et al., 2006	Effects of sun ginseng on subjective quality of life in cancer patients: A double-blind, placebo-controlled pilot trial	*Journal of Clinical Pharmacy and Therapeutics*	**Ineligible formulation:** The intervention consisted of an orally administered processed ginseng product, but the report did not establish that it originated from an infusion, decoction, or eligible aqueous-extraction process. The solid dosage form was not itself the reason for exclusion.
18	You et al., 2009	Effect of extracts from indigowood root (*Isatis indigotica* Fort.) on immune responses in radiation-induced mucositis	*Journal of Alternative and Complementary Medicine*	**Ineligible administration procedure:** The preparation was used primarily as an oral gargle rather than as a prespecified orally ingested intervention.
19	Marucci et al., 2017	Double-blind randomized Phase III study comparing a mixture of natural agents versus placebo in the prevention of acute mucositis during chemoradiotherapy for head and neck cancer	*Head & Neck*	**Ineligible route:** The multi-agent preparation was used as a topical oral treatment rather than as an ingested herbal intervention.

20	Hirvonen et al., 2006	Consumption of antioxidant-rich beverages and risk for breast cancer in French women	*Annals of Epidemiology*	**Ineligible study design:** Observational epidemiological study rather than a randomized clinical trial.
21	Yaghobi et al., 2021	Combination of traditional herbal medicine for the treatment of cancer-induced anorexia/cachexia: A pilot, randomized, double-blinded and placebo-controlled clinical trial	*Journal of Herbal Medicine*	**Insufficient formulation information:** The report did not provide sufficient evidence that the administered multi-ingredient herbal product originated from an infusion, decoction, or eligible aqueous-extraction process.
22	Han et al., 2016	Chinese herbal medicine as maintenance therapy for improving the quality of life of patients with advanced non-small-cell lung cancer	*Complementary Therapies in Medicine*	**Ineligible study design:** Treatment allocation did not satisfy the prespecified randomized-trial requirement.
23	Hao et al., 2017	Cardioprotective effect of *Platycodon grandiflorum* in patients with early breast cancer receiving anthracycline-based chemotherapy: Study protocol for a randomized controlled trial	*Trials*	**Unavailable eligible results:** Study protocol without published clinical results from the planned randomized trial.
24	Lee et al., 2014	Adjunctive traditional Chinese medicine therapy improves survival in patients with advanced breast cancer: A population-based study	*Cancer*	**Ineligible study design:** Observational population-based study rather than a randomized clinical trial.
25	Guglielmo et al., 2020	A randomized, double-blind, placebo-controlled Phase II study to evaluate the efficacy of ginseng in reducing fatigue in patients treated for head and neck cancer	*Journal of Cancer Research and Clinical Oncology*	**Insufficient formulation information:** The report did not establish that the encapsulated ginseng preparation originated from an infusion, decoction, or eligible aqueous-extraction process. The capsule dosage form was not itself the reason for exclusion.
26	Jatoi et al., 2003	A Phase II trial of green tea in the treatment of patients with androgen-independent metastatic prostate carcinoma	*Cancer*	**Ineligible study design:** Single-arm Phase II trial without a randomized comparator.

27	Perez et al., 2010	A Phase 1B dose-escalation trial of *Scutellaria barbata* (BZL101) for patients with metastatic breast cancer	*Breast Cancer Research and Treatment*	**Ineligible study design:** Single-arm Phase 1B dose-escalation study without a randomized comparator.
28	ClinicalTrials.gov, 2013	A pilot study of Teng-Long-Bu-Zhong-Tang-based herbal therapy in combination with chemotherapy in patients with metastatic colorectal cancer	ClinicalTrials.gov, NCT01975454	**Unavailable eligible results:** Trial-registry record without an associated publication reporting eligible randomized clinical results.
29	ClinicalTrials.gov	A pilot safety, feasibility, efficacy and correlative Phase I/II study assessing Herba Scutellariae Barbatae for metastatic breast cancer	ClinicalTrials.gov, NCT00028977	**Unavailable eligible results:** Trial-registry record without an associated publication reporting eligible randomized clinical results.

Abbreviations: RCT, randomized controlled trial. Reports and trial-registry records were excluded after full-text eligibility assessment according to the prespecified population, intervention, preparation method, route of administration, study design, and clinical-outcome criteria. One primary reason for exclusion was assigned to each record. Eligibility of solid oral products was determined by documented evidence that the administered intervention originated from an infusion, decoction, or eligible aqueous-extraction process; capsules, granules, powders, and other solid dosage forms were not excluded solely because of their final presentation. When the study report did not describe the extraction solvent or provide sufficient information to establish correspondence with the operational intervention definition, the record was classified as having insufficient formulation information. The Kitchenham-based assessment was used exclusively for descriptive purposes and was not applied as an eligibility threshold, risk-of-bias judgment, or substitute for the RoB 2 assessment.

Table S3. GRADE Summary of Findings—Outcome-level certainty-of-evidence assessment for the eight included randomized controlled trials.

**Intervention**	**Population**	**Outcome**	**Participants (studies)**	**Effect estimate**	**Follow-up**	**RoB 2**	**GRADE certainty**	**Clinical interpretation**
Gami-guibi-tang (GGBT)	Gastrointestinal, breast, and lung cancers	Sleep quality (ISI score)	30 (1 RCT)	Mean change: −5.5 versus +0.1; $p < 0.001$; 95% CI NR	2 weeks	Some concerns	⊕○○○ Very Low	Possible short-term benefit; evidence was derived from a small, open-label pilot trial without a placebo control. The MCID and 95%

								CI were not reported.
Gami-guibi-tang (GGBT)	Gastro-intestinal, breast, and lung cancers	Cancer-related fatigue (BFI score)	30 (1 RCT)	Mean change: −0.8 versus 0.0; $p = 0.002$; 95% CI NR	2 weeks	Some concerns	⊕◯◯◯ Very Low	Possible short-term benefit, but its magnitude and clinical importance remain uncertain because the MCID and 95% CI were not reported.
Rikkun-shito	Lung cancer treated with cisplatin-based chemotherapy	Chemotherapy-induced anorexia (reduction in caloric intake)	31 (1 RCT)	18% versus 25% reduction; $p = 0.025$; 95% CI NR	14 days	Some concerns	⊕◯◯◯ Very Low	Possible modest attenuation of the reduction in caloric intake. The per-protocol analysis, small sample, short follow-up, and absence of a 95% CI limit interpretation.
Rikkun-shito	Lung cancer treated with cisplatin-based chemotherapy	Plasma acylated ghrelin recovery	31 (1 RCT)	Greater recovery by day 5 with Rikkun-shito versus control; $p = 0.025$; 95% CI NR	14 days	Some concerns	⊕◯◯◯ Very Low	This was a biological endpoint. The clinical relevance of the observed ghrelin recovery has not been established.
Holy basil (*Ocimum tenuiflorum*) tea	Differentiated thyroid cancer treated with RAIT	Anxiety (STAI score)	NR (1 RCT)	Significant reduction versus control; $p < 0.05$; numerical effect estimate and 95% CI NR	4 days	Some concerns	⊕◯◯◯ Very Low	Preliminary evidence from a very short intervention with incompletely reported blinding procedures. The magnitude and

								clinical importance of the effect could not be established.
Holy basil (*Ocimum tenuiflorum*) tea	Differentiated thyroid cancer treated with RAIT	Salivary-gland function (washout ratio)	NR (1 RCT)	Better preservation versus control; $p < 0.05$; numerical effect estimate and 95% CI NR	4 days	Some concerns	⊕○○○ Very Low	Preliminary evidence based on an objective scintigraphic outcome. The very short follow-up and absence of trial-specific phytochemical characterization limit interpretation.
Alcea digitata–Malva sylvestris infusion	Head and neck cancer with radiation-induced xerostomia	Xerostomia and quality of life (EORTC QLQ-H&N35)	NR (1 RCT)	Significant improvement versus artificial saliva; $p < 0.05$; numerical effect estimate and 95% CI NR	4 weeks	High risk	⊕○○○ Very Low	Symptomatic improvement was reported, but the open-label design, active comparator, incomplete effect estimates, and high risk of bias substantially reduce confidence in the result.
Annona muricata aqueous-leaf-extract-derived preparation	Colorectal cancer, stages I–IV	Nutritional status	30 randomized; 28 analyzed (1 RCT)	No statistically significant between-group difference; numerical effect estimate and 95% CI NR	8 weeks	Some concerns	⊕○○○ Very Low	A specific nutritional benefit was not demonstrated. The small sample, incomplete-case analysis, and absence of a precise effect estimate

								limit confidence in the finding.
Annona muricata aqueous-leaf-extract-derived preparation	Colorectal cancer, stages I–IV	*Ex vivo* cytotoxic activity (exploratory surrogate endpoint)	30 randomized; 28 analyzed (1 RCT)	Greater cytotoxic activity in participant serum than with placebo; numerical effect estimate and 95% CI NR	8 weeks	Some concerns	⊕◯◯◯ Very Low	This exploratory surrogate laboratory finding does not constitute evidence of direct clinical antitumor efficacy.
Malva sylvestris–Althaea digitata preparation	Prostate cancer treated with pelvic radiotherapy	Anal burning (VAS score)	NR (1 RCT)	Approximately 2.3-point lower VAS score versus placebo; 95% CI NR	5 weeks	Some concerns	⊕◯◯◯ Very Low	A possible subjective symptomatic benefit was observed. Its clinical importance remains uncertain because the MCID and 95% CI were not reported.
Malva sylvestris–Althaea digitata preparation	Prostate cancer treated with pelvic radiotherapy	Diarrhea severity (RTOG grading)	NR (1 RCT)	Between-group difference in RTOG grading was not statistically significant; numerical effect estimate and 95% CI NR	5 weeks	Some concerns	⊕◯◯◯ Very Low	No statistically significant benefit was demonstrated according to objective RTOG grading; evidence for preventing radiation-related gastrointestinal toxicity remains inconclusive.
Jing Si herbal tea (JSHT)	Gynecologic cancer with	Cancer-related fatigue (BFI-T)	21 enrolled; 19 analyzed (1 RCT)	No statistically significant between-group	6 weeks; placebo-controlled	Some concerns	⊕◯◯◯ Very Low	No specific between-group benefit was demonstrated.

	moderate-to-severe cancer-related fatigue			difference in improvement; numerical effect estimate and 95% CI NR	comparison during the first 3 weeks			Within-group improvements cannot establish efficacy. The very small sample, incomplete analysis, and short placebo-controlled phase limit confidence in the result.
Jing Si herbal tea (JSHT)	Gynecologic cancer with moderate-to-severe cancer-related fatigue	Quality of life (FACT-G7)	21 enrolled; 19 analyzed (1 RCT)	No statistically significant between-group difference in improvement; numerical effect estimate and 95% CI NR	6 weeks; placebo-controlled comparison during the first 3 weeks	Some concerns	⊕◯◯◯ Very Low	No specific between-group benefit was demonstrated. The very small sample, incomplete analysis, short placebo-controlled phase, and imprecision limit confidence in the result.
Renshen Yangrong Tang (RSYRT) herbal extract granules	Cancer survivors with clinically relevant fatigue	Cancer-related fatigue (MDASI fatigue score)	83 analyzed (41 RSYRT; 42 low-dose huangqi control) (1 RCT)	Time-by-group estimate: −0.61 (SE 0.10), $p < 0.0001$. A reduction of ≥2 points was reported in 90.2% versus 52.4% of participants. At week 4, mean change was −2.66 versus −1.36; standardized effect	6 weeks	Some concerns	⊕◯◯◯ Very Low	RSYRT was associated with a greater short-term reduction in fatigue than low-dose huangqi. However, the active herbal comparator, unclear blinding and allocation-concealment procedures, relatively small sample, and absence of a

				size, d = 1.0; 95% CI NR				reported 95% CI limit certainty and generalizability.

GRADE certainty rating: ⊕◯◯◯ Very Low indicates very little confidence in the effect estimate; the true effect is likely to be substantially different from the reported estimate. Outcome-level certainty was downgraded because of: (1) risk of bias—most trials showed some concerns or high risk of bias according to RoB 2; (2) imprecision—estimates were derived from single, generally small trials, and 95% confidence intervals were frequently unreported or insufficiently precise; and, where applicable, (3) indirectness—some outcomes were surrogate, exploratory, or of uncertain clinical relevance. The certainty assessment was performed independently of the feasibility of meta-analysis. Meta-analysis was not undertaken because substantial clinical and methodological heterogeneity precluded meaningful quantitative pooling across the eight included trials. No two trials evaluated the same combination of herbal intervention, cancer population, treatment context, and principal outcome measure. Abbreviations: BFI, Brief Fatigue Inventory; BFI-T, Brief Fatigue Inventory–Taiwan Form; CI, confidence interval; EORTC QLQ-H&N35, European Organisation for Research and Treatment of Cancer Quality of Life Questionnaire–Head and Neck Module; FACT-G7, Functional Assessment of Cancer Therapy–General 7-item version; GGBT, Gamiguibi-tang; ISI, Insomnia Severity Index; JSHT, Jing Si herbal tea; MCID, minimal clinically important difference; MDASI, MD Anderson Symptom Inventory; NR, not reported; QoL, quality of life; RAIT, radioactive iodine therapy; RCT, randomized controlled trial; RoB 2, revised Cochrane Risk of Bias tool for randomized trials; RSYRT, Renshen Yangrong Tang; RTOG, Radiation Therapy Oncology Group; SE, standard error; STAI, State–Trait Anxiety Inventory; VAS, visual analog scale.

Table S4. Domain-level risk-of-bias assessment of the principal outcomes of the eight included randomized controlled trials according to the Cochrane Risk of Bias 2 (RoB 2) tool.

**Study**	**Randomization process**	**Deviations from intended interventions**	**Missing outcome data**	**Measurement of the outcome**	**Selection of the reported result**	**Overall judgment**	**Main reason**
Lee et al., 2018	Low risk	Some concerns	Low risk	Low risk	Low risk	Some concerns	The open-label design, absence of a placebo, and incomplete information regarding adherence to the assigned interventions may have influenced the subjective sleep and fatigue outcomes.
Yoshiya et al., 2020	Low risk	Some concerns	Some concerns	Low risk	Low risk	Some concerns	The per-protocol analysis, post-randomization exclusions, crossover design, and absence of a placebo raised concerns regarding deviations from the intended interventions and missing outcome data.

Nomura et al., 2023	Low risk	Some concerns	Low risk	Some concerns	Low risk	Some concerns	Participant and investigator blinding was not clearly reported. Knowledge of the assigned intervention may have influenced the subjective anxiety outcome, although salivary-gland function was assessed objectively.
Heydarirad et al., 2017	Some concerns	High risk	Low risk	Some concerns	Low risk	High risk	The open-label design, subjective xerostomia and quality-of-life outcomes, absence of a placebo infusion, and insufficiently reported randomization procedures created a high risk that knowledge of the assigned intervention influenced the reported effects.
Indrawati et al., 2017	Low risk	Low risk	Some concerns	Some concerns	Low risk	Some concerns	Two participants withdrew, no missing-data imputation method was reported, and exploratory laboratory outcomes were analyzed among the available participants.
Moeini et al., 2018	Low risk	Low risk	Some concerns	Low risk	Low risk	Some concerns	Withdrawals occurred during follow-up, and the handling of missing outcome data was incompletely reported.
Li et al., 2025	Some concerns	Low risk	Some concerns	Low risk	Some concerns	Some concerns	The random-sequence generation and allocation-concealment procedures were not clearly reported. Nineteen of 21 enrolled participants were analyzed without a reported imputation method, and the prespecified analysis procedures were insufficiently documented.
Xu et al., 2020	Some concerns	Some concerns	Some concerns	Some concerns	Some concerns	Some concerns	The random-sequence generation, allocation-concealment, and blinding procedures were not clearly reported. Fatigue was a participant-reported outcome, and the comparator was low-dose huangqi rather than an inert placebo. Eighty-three participants were included in the reported analysis, but the handling of missing outcome data and the

							prespecified analysis procedures were insufficiently documented.

RoB 2, revised Cochrane Risk of Bias tool for randomized trials. Risk-of-bias judgments were assigned independently by two reviewers according to RoB 2 guidance and refer to the principal clinical outcome or outcomes evaluated in each trial. Overall judgments were derived from the domain-level assessments rather than from the descriptive Kitchenham-based methodological instrument. Low risk indicates that bias was unlikely to materially affect the reported result; some concerns indicate the presence of methodological or reporting limitations that reduce confidence in the result; and high risk indicates important limitations likely to affect the validity of the reported result. The absence of participant or investigator blinding was interpreted in relation to the nature of the outcome and was considered particularly relevant for subjective patient-reported outcomes. "Not reported" information was not assumed to have been adequately implemented.

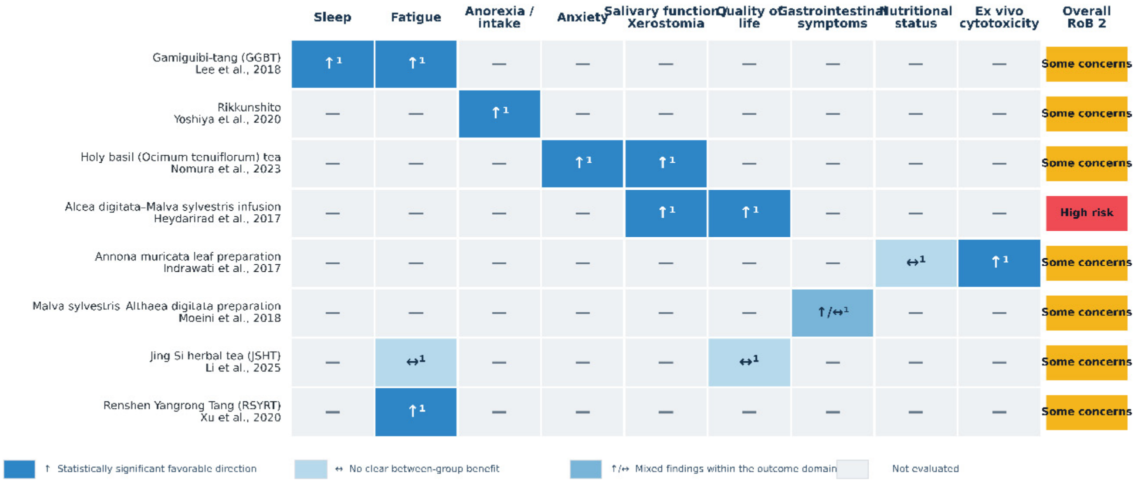

Figure S1. Intervention-by-outcome evidence-gap heatmap for the eight included randomized controlled trials. Dark-blue cells containing an upward arrow (↑) indicate a statistically significant favorable direction of effect; light-blue cells containing a horizontal arrow (↔) indicate no clear between-group benefit or maintenance without demonstrated superiority; cells containing both symbols (↑/↔) indicate mixed findings within the same outcome domain; and light-gray cells containing a dash (—) indicate that the outcome was not evaluated. Empty evidence domains should not be interpreted as evidence of no effect. The superscript number indicates the number of RCTs contributing evidence to each intervention–outcome relationship (^{1} = one RCT). All evaluated relationships were supported by a single trial and were rated as very-low-certainty evidence. The final column presents the overall RoB 2 judgment: yellow indicates some concerns and red indicates high risk of bias. Renshen Yangrong Tang was associated with a statistically significant short-term reduction in fatigue compared with low-dose huangqi, although the active herbal comparator and methodological limitations reduce certainty in the finding. For *Annona muricata*, nutritional status did not differ significantly between groups, whereas cytotoxic activity was assessed only through an exploratory *ex vivo* experiment and should not be interpreted as evidence of clinical antitumor efficacy. For the *Malva sylvestris–Althaea digitata* preparation, a reduction in subjective anal-burning scores was reported, but the between-group difference in objective RTOG grading was not statistically significant; this outcome domain is therefore presented as having mixed findings. Abbreviations: RCT, randomized controlled trial; RoB 2, revised Cochrane Risk of Bias tool for randomized trials; RSYRT, Renshen Yangrong Tang; RTOG, Radiation Therapy Oncology Group.

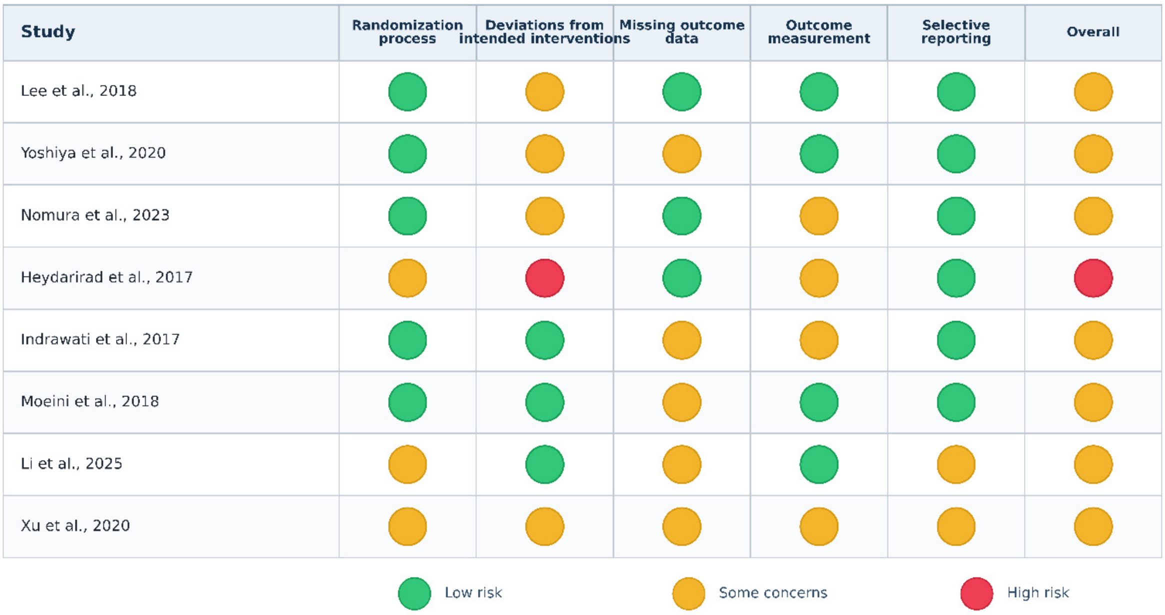

Figure S2. Risk-of-bias assessment of the principal outcomes of the eight included randomized controlled trials according to the revised Cochrane Risk of Bias tool for randomized trials (RoB 2). Green circles indicate low risk of bias, yellow circles indicate some concerns, and red circles indicate high risk of bias. Xu et al., 2020 was judged as having some concerns across all domains because the random-sequence generation, allocation-concealment, blinding, handling of missing outcome data, and prespecified analysis procedures were insufficiently documented; moreover, fatigue was participant-reported and the comparator was low-dose huangqi rather than an inert placebo. Overall judgments were derived from the domain-level assessments and were not based on the descriptive Kitchenham instrument. Abbreviation: RoB 2, revised Cochrane Risk of Bias tool for randomized trials.

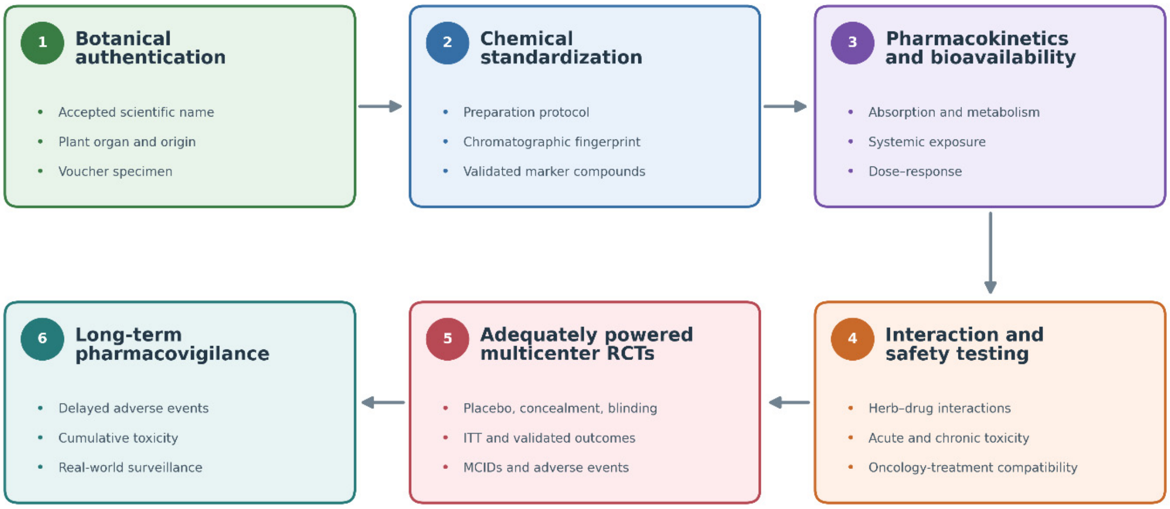

Figure S3. Translational roadmap for the clinical development of orally administered herbal preparations in supportive cancer care. The proposed framework emphasizes sequential botanical authentication, chemical standardization, pharmacokinetic and bioavailability assessment, herb–drug interaction and safety testing, adequately powered multicenter randomized controlled trials, and long-term pharmacovigilance. Each stage addresses evidence gaps identified across the included trials and should be completed before broader clinical implementation. Abbreviations: ITT, intention-to-treat; MCID, minimal clinically important difference; RCT, randomized controlled trial.

https://doi.org/10.3390/xxxxx
